# Caries in orphan children: prevalence and determinants—a systematic review and meta-analysis

**DOI:** 10.1186/s12903-024-04125-9

**Published:** 2024-03-25

**Authors:** Ayat Gamal-AbdelNaser, Mennat Allah Ashraf A.Elsabour, Nagwa Mohamed Ali Khattab

**Affiliations:** 1 Department of Oral Medicine and Periodontology, Faculty of Oral and Dental Medicine, Ahram Canadian University, 4th Industrial Zone, Banks Complex, 6th of October City, Giza Egypt; 2 Pediatric and Community Dentistry Department, Ahram Canadian University, 6th of October City, Giza Egypt; 3 Pediatric Dentistry and Dental Public Health Department, Ain Shams University, Cairo, Egypt

**Keywords:** AHRQ, Caries risk factors, DMF, Orphanage, Vulnerable group

## Abstract

**Background:**

Orphan children represent a category of children who lost their family support. Their health status is poorer when compared to their parented counterparts. As the most prevalent disease in the world, dental caries is expected to affect orphans greatly. Being vulnerable, health status of orphan children must be monitored and studied; so that health improvement plans would be formulated. Therefore, this systematic review focuses on the extent of the dental caries problem among institutionalized orphan children and its determinants.

**Methods:**

The review has two outcomes: comparing caries experience of institutionalized children to their parented counterparts, and reviewing the determinants of caries in the exposure group. Two systematic searches (one for each outcome) were run on MedLine via PubMed, Cochrane library, LILACS, Egyptian knowledge bank (EKB) and Google Scholar; beside hand search and searching grey literature.

**Results:**

The searches yielded 17,760, followed by 16,242 records for the first and second outcomes respectively. The full text was screened for 33 and 103 records for the two outcomes respectively; after translating non-English reports. Finally, the review included 9 records to address the first outcome and 21 records for the second. The pooled results showed that the exposure group may show slightly poorer caries experience regarding permanent teeth (pooled mean difference of DMF = 0.09 (-0.36, 0.55)); but they have a much poorer caries experience regarding primary teeth health (pooled mean difference of dmf = (0.64 (-0.74, 2.01)). Meta-analysis of the caries determinants showed that institutionalization increases the risk of caries by 19%. Gender showed slight effect on caries risk with males being more affected; while primary teeth revealed higher risk of caries when compared to permanent teeth.

**Conclusion:**

Limited by the heterogeneity and risk of bias of the included studies, meta-analyses concluded that institutionalized orphan children have higher risk of caries. Yet, the institutionalization circumstances were not well-documented in all the included studies. So, the complete picture of the children’s condition was not possibly sketched.

**Trial registration:**

Protocol has been registered online on the PROSPERO database with an ID CRD42023443582 on 24/07/2023.

**Supplementary Information:**

The online version contains supplementary material available at 10.1186/s12903-024-04125-9.

## Background

An orphan child is a child who lost family support early in life. This loss is reflected in many aspects of the orphan children’s lives, including their health and overall well-being. Generally, children tend to imitate their parents [1], and to learn health and hygiene practices from them. Furthermore, parents have a major role in supervising their children's health. Consequently, the absence of parents is expected to significantly influence the children's health, particularly their oral health [2].

Concerning oral health, dental caries represents the most prevalent disease worldwide, especially among children [3]. Beside its high prevalence, dental caries was found to considerably compromise the individual’s quality of life in different ways [4]. According to a recent systematic review [5], dental caries is proven to negatively influence the oral health-related quality of life (OHRQoL) of children generally. Aiming to control such a disease and its negative consequences, many studies were conducted to estimate its actual prevalence and distribution among different populations and to determine different factors that modulate the disease among these populations [3, 6–9].

Among these studies, some targeted institutionalized orphan children as a special vulnerable group [10–13]. Their vulnerability is attributed to financial limitations, crowding, improper caretaker-to-child ratio, absence of proper supervision of the children, and poor nutritional state [14–16].

Individual studies investigated caries experience in this vulnerable group [10–13]. This concern was adopted by studies since the 1930s [17–19]; and extended to be at the summit of research interest through years to date [20, 21]. Besides, it was investigated in a huge number of countries in almost all the continents among which are: the USA [17], Haiti [22], Brazil [23], Mexico [16], United kingdom [24], Germany [25, 26], Portugal [27], Hungary [28], Romania [29], Russia [30, 31], China [32], India [33–35], Indonesia [36], Pakistan [2], Turkey [37], Yemen [12], Saudi Arabia [38], Iraq [39], Iran [40], Egypt [20], South Africa [41], Tanzania [42] and Uganda [43].

However, to the best of our knowledge, the results of these enormous numbers of studies have not been gathered to calculate a pooled estimate of the degree of suffering of such a population. Furthermore, the results of these individual studies fluctuate greatly from studies showing higher prevalence in this population [10, 12, 44] to other studies revealing institutionalized children to be more protected than parented ones [45–47]. In addition, the factors influencing dental caries in the specified population is not well established in literature. Therefore, to date, it is not possible to plan special healthcare guidelines and formulate clear and strict recommendations for optimal dental healthcare to these children.

Therefore, to estimate the gap between caries experience between institutionalized orphan children and children who are sheltered by their parents, this systematic review was performed. It further aims to review the determinants which modify the orphan children's caries experience.

## Methods

The current systematic review is concerned with answering two research questions:

1. In children, does the dental caries experience differ significantly among institutionalized orphans, when compared to their parented counterparts?
2. Regarding institutionalized orphan children, is the dental caries experience modified by any determinants?

### Eligibility criteria

For the two outcomes, studies were considered eligible when they followed observational study design, and included participants who are institutionalized orphan children, with age range from 6 months to 18 years, and are medically free from any systemic or genetic disorders.

For the first outcome, the studies had to include a comparator group; where the institutionalized children should be compared to parented children living with their families. While for the second outcome, a comparator was not a must.

Regarding the outcomes of the included studies, the study had to report caries experience assessed by caries index (DMFT/ deft/ dmft) to be included in the answer of the first review question. Our review adopted the total DMF/ def/ dmf (DMFT/ deft/ dmft) scores according to the WHO specifications [48, 49] where total DMF score is used for the permanent dentition, total DMF/def score for the mixed dentition, and total dmf score for primary dentition. As much as this criterion restricted the inclusion of reports in the current review, it safeguarded against inconsistent results that would have not been possibly combined statistically or qualitatively. On the other hand, for a study to be included in the answer of the second question, it should report any caries determinant.

### Information sources

A detailed search strategy was followed to search on MedLine via PubMed, Cochrane library, LILACS, Egyptian knowledge bank (EKB) and Google Scholar. Search was extended to grey literature (open grey) and hand searching the reference lists of the retrieved studies. The search had no time or language restriction. The search strategies used for the search of the two outcomes are listed in full details in (Additional file 1).

### Selection process

The search was run on the afore-specified databases by two authors (AG and MAA) in duplicates. The retrieved studies were de-duplicated and managed using Mendeley (Version 1.19.8) reference manager software. Then, the results of the search were screened by title and abstract independently to check for their eligibility based on the prespecified criteria. When decided eligible or when eligibility is unclear, the full texts of the studies were screened, and eligibility was decided by the two reviewers independently. After each step, the review team met to check the consistency of the results of the two reviewers. Any disagreements in the decisions of the two review authors were resolved by discussion, and by consulting the guarantor (NMAK). Whenever the full text of a record was not retrieved, the reviewers contacted the authors, journal and/or publisher twice.

### Data collection process

Data of the included studies were extracted in a pre-set standardized data extraction table by two (AG and MAA) reviewers in duplicates. As usual, disagreements were resolved by discussion and by consulting the guarantor. Whenever some details were unclear regarding a certain record, the corresponding author was contacted twice to clarify the ambiguity.

### Data items

In each included study, we reported the country, city, and the type of orphanage in which the study was held. Regarding the participants of each study, their number, age range, and gender were reported. Then, the outcomes were reported just as mentioned in the study in the form of DMF and dmf for the first review outcome (mainly as mean and standard deviation). For the second outcome, caries determinants assessed in each study were listed and the results were discussed.

### Study risk of bias (RoB) assessment

The risk of bias of each included study was assessed using the Agency for Healthcare Research and Quality tool (AHRQ) for the cross-sectional studies. AHRQ was the most suitable tool as it was recommended for assessing non-RCT articles handling dental caries among children [50].

AHRQ consists of 11 items; each is awarded one score (1 = yes; 2 = not mentioned; 3 = unclear). The AHRQ items include: defining data source, listing eligibility criteria, indicating the study’s time period, indicating if the study sample was representative, indicating the masking of the evaluator, using valid assessment method, explaining patient’s exclusion from analysis, controlling the confounders, handling missing data, mentioning the patient’s response rate, and clarifying follow up when applicable [51].

Using the above-mentioned tool, the assessment was performed by two (AG and MAA) reviewers independently. The decision of the reviewers regarding the risk of bias assessment was justified using verbatim quotes from the assessed study. Any conflicts in the decision were resolved by discussion, and by consulting reviewer (NMAK).

### Effect measures and synthesis methods

When more than three included studies were measuring the same outcome using consistent measuring methods, the results of the studies were combined in meta-analysis. The unit of analysis was the participant. Each outcome was analyzed separately. A pooled estimate of the difference in caries experience between institutionalized orphan children and parented children was calculated through meta-analyzing results of studies reporting caries experience as a continuous outcome (mean and standard deviation of DMF/def/dmf). For this outcome, two analyses were performed; one for the caries experience of primary teeth (dmf) and another for that of permanent teeth (DMF).

Furthermore, in some studies, caries was represented in the form of “caries prevalence”, where the number of affected children were reported versus the unaffected children. The child was considered to have dental caries if reported to have a total DMF/def/dmf scores of more than zero [3]. In this case, the reported determinants were assessed as being risk factors for caries by combining studies that reported the caries prevalence to determine the risk ratio of the determinant.

Heterogeneity was tested through the Chi-squared test and I-squared test. An I^2^ of less than 40% indicates inconsiderable heterogeneity and so fixed-effect model was used; otherwise, random-effects model was selected [52].

As highlighted, meta-analysis was performed for outcomes which were measured consistently by more than three included studies. When meta-analysis was performed, all continuous outcomes were analyzed using a weighted mean difference with 95% CI.; while dichotomous outcomes were presented as risk ratio.

If the quantitative analysis was not possible, a qualitative summary was reported in a narrative way. Meta-analyses were conducted with the help of RevMan 5.4 software.

#### Reporting bias

When the number of studies included in one outcome was more than 10, publication bias was tested by funnel plot. Selective reporting bias was checked by exploring the study protocol if published.

#### Certainty assessment

The level of evidence of all outcomes was rated using the GRADE guidelines into high, moderate, low, and very low.

#### Reporting guidelines

The review has been performed and drafted following the PRISMA guidelines and checklist [53].

## Results

The review addressed two questions regarding: (i) the caries experience among orphan children in comparison to parented children, and (ii) the determinants that modify the caries experience in institutionalized orphan children.

### Study selection

The results of the search yielded 17,760 and 16,242 records for the first and second outcomes respectively. After de-duplication and screening by title and abstract, full texts were screened for eligible studies. Full texts of some records [25, 26, 30, 35, 36, 54–66] were not possibly retrieved even after contacting the authors, journals and publishers.

The reviewers screened the full text of 33 records for the first outcome, and 103 for the second outcome. Records that were not reported in English were professionally translated from Hungarian, Portuguese, Russian, Chinese, Korean, Lithuanian, Persian and Ukrainian.

The ineligible records were excluded after justifying the grounds of their exclusion (Additional file 2). The main causes of exclusion encompassed not excluding medically compromised children, being a review article, using ineligible assessment method, not assessing caries and not including orphans.

**Fig. 1:**
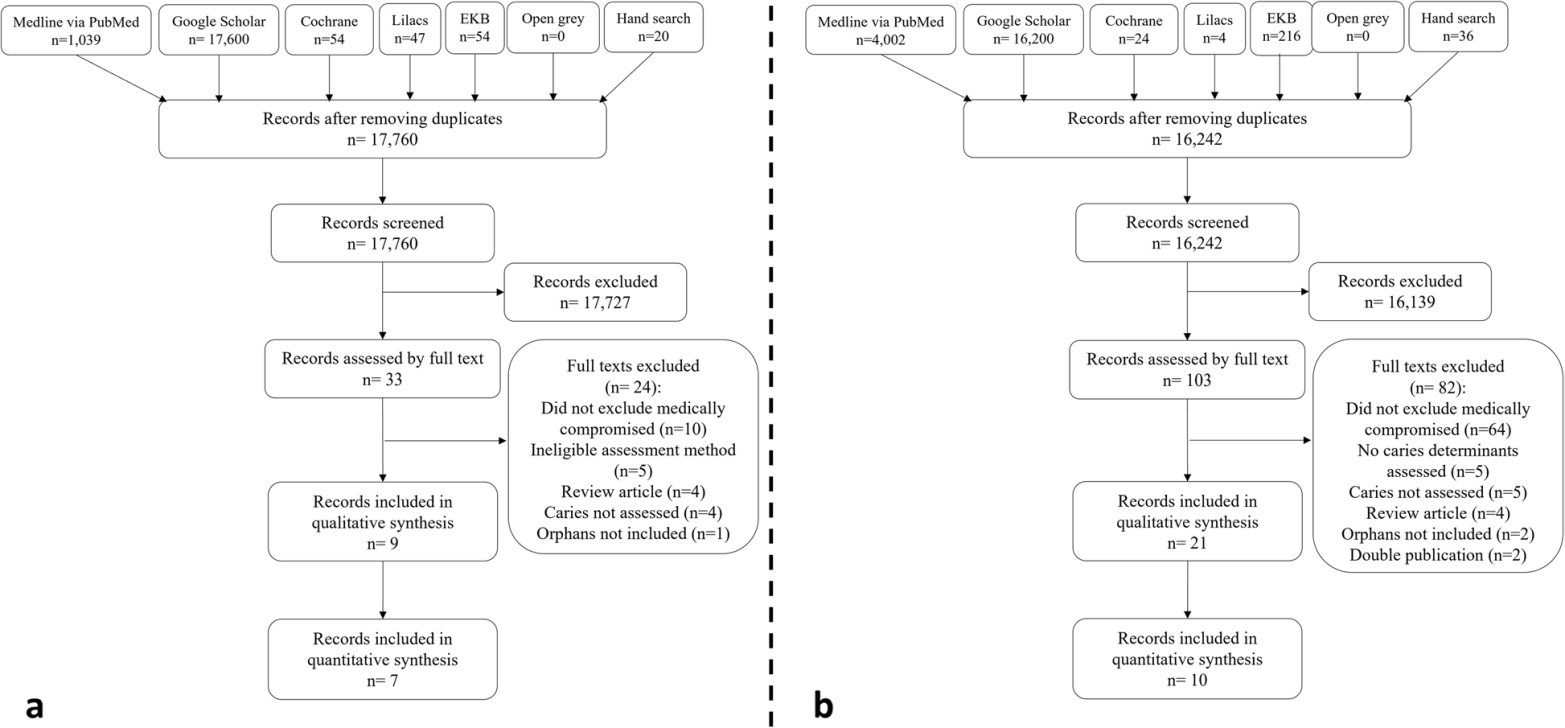
Flowcharts for the review steps of the two outcomes; **a** for the studies comparing caries experience of institutionalized compared to parented children, and **b** for the studies reporting caries experience and any of its determinants in institutionalized children

Eventually, the review included 9 records to address the caries experience of orphans compared to parented children [10, 12, 20, 32, 44, 46, 47, 67, 68], and 21 records for the caries determinants in orphan children [10, 12, 20, 32, 40, 44–47, 67–78] (Fig. 1).

When a dissertation and the article derived from it were both available, they were assessed as one record where missing data in one document was retrieved from the other [75, 79].

### Study characteristics

Nine studies assessed the caries experience of orphan children in comparison to parented counterparts (Table 1). Adding to the pre-discussed nine studies, twelve more were eligible to the second outcome (Table 2).

**Table 1:** Characteristics of studies answering the first outcome: Caries experience (DMF/dmf) in Institutionalised vs non-institutionalized

	**Record**	**Age (years)**	**Sex**	**Country-City**	**Sample size**	**Orphanage type**	**Mean DMF/def (or dmf)**		**Conclusion**
							**Institution-alised (O)**	**Controls (C)**	
**1**	**(Agarwalla et al., 2022)** [47]	7–11	Both: 25 males & 25 females in each group	India	**100:** 50 per group	Data not available	DMF = 1.54 ± 2.09	DMF = 2.52 ± 3.04	DMF of **O < C not** significantly (*P* = 0.064)
**2**	**(Al-maweri et al., 2014)** [12]	6–15	Males only (404)	Sana’a-Yemen	**404:** 202 per group	NGO	dmft = 2.28 ± 2.37	dmft = 3.82 ± 2.57	dmft** C > O** **significantly** *P* < 0.001
							DMFT = 2.06 ± 1.94	DMFT = 1.77 ± 1.58	**O > C not** significantly (*P* > 0.05)
**3**	**(Gaytry, 2018)** [67]	8–14	Data not available	Namakkal district- India	**400:** 200 per group	Data not available	**No. of participants:** DMF ≤ 3:163 DMF > 3:37	**No. of participants:** DMF ≤ 3:138 DMF > 3:62	**C > O** **Significantly** *P* = 0.004
**4**	**Khattab & Abd-ElSabour, 2023)** [20]	6–12	Both: 73 males & 83 females	Giza- Egypt	**156:** 52 per group	NGO & Gov orphanages	**def** NGO = 1.69 ± 2.58 Gov = 0.41 ± 0.89	**def = ** 0.85 ± 1.79	def **NGO > C > Gov** **Significantly** *P* < 0.01
							**DMF** NGO = 1.86 ± 2.96 Gov = 1.80 ± 2.54	**DMF** = 0.75 ± 1.29	**DMF** **NGO > Gov > C** **Significantly** *P* = 0.025
**5**	**(Mehta et al., 2020)** [46]	12–15	Both: 235 males & 115 females	Pune district- India	**350:** 175 per group	Data not available	**DMFT = ** 1.93 ± 1.21	**DMFT = ** 2.66 ± 1.27	**DMFT** **C > O** **Significantly** *P* < 0.001
**6**	**(Meshki et al., 2022)** [10]	7–12	Both: detailed numbers not available	Mashhad- Iran	**444:** 222 per group	Gov	**dmft** = 9.01 ± 3.85	**dmft** = 5.2 ± 3.4	**dmft O > C** **Significantly** *P* = 0.003
							**DMFT** = 3.36 ± 1.86	**DMFT = ** 2.1 ± 1.8	**DMFT O > C** **not** significantly *P* = 0.6
**7**	**(Pavithran et al., 2009)** [44]	12–15	Both: 213 males & 207 females	Bengaluru city- south India	**420:** 210 per group	Data not available	**DMFT** = 0.76 ± 1.26	**DMFT** = 0.64 ± 1.34	**DMFT O > C** **not** significantly *P* = 0.370
**8**	**(Rimaviciute et al., 2019)** [68]	12 and 15	Both: 64 males & 46 females	South Lithuania	**110:** 55 per group	Social care? (government?)	12-year-old: DMFT = 2.0 ± 3.25 15-year-old DMFT = 3.0 ± 3.0	12-year-old: DMFT median = 1 15-year-old DMFT median = 3	**O > C** **Significantly** *P* < 0.01
**9**	**(Xu et al., 2021)** [32]	3–5, 12–15	Both: 523 males & 399 females	Fuyang city- China	**922:** 332 **(O)** vs 590 **(C)**	Data not available	dmf = 4.41 ± 2.6	dmf = 3.29 ± 2.05	**dmf: O > C** **Significantly**
							DMF = 1.28 ± 1.26	DMF = 1.11 ± 1.24	**DMF: O > C** **Significantly**

*C* Control group, *Gov* governmental, *NGO* Non-governmental organization; O: Orphanage group

**Table 2:** Characteristics of the rest of the studies addressing the second outcome: Caries experience in institutionalized children

	**Record**	**Age (years)**	**Sex**	**Country-City**	**Sample size**	**Orphanage type**	**Caries experience**
**1**	**(Abedassar et al., 2022)** [76]	6–18 (6–12, 13–18)	Both: 180 males & 176 females	Kerman Province- southeast Iran	**356**	Data not available	6**–12-year-old:** dmft = 4.13 ± 3.80 **13–18-year-old**: dmft = 1.26 ± 1.65
							**6–12-year-old:** DMFT = 1.73 ± 1.84 **13–18-year-old:** DMFT = 4.98 ± 3.60
**2**	**(Chandran, 2017)** [79]	12–17	Both: 159 males & 110 females	Bengaluru city- India	**269**	Data not available	DMFT mean = 3.55
**3**	**(Kavayashree & Babu, 2019)** [74]	6–14	Both: 63 males & 39 females	Hassan district - south India	**100**?? But data of 103?	NGO	(deft) = 0.69 ± 1.25 males = 0.69 ± 0.15 Females = 0.68 ± 0.23 (p = 0.977) DMFT = 0.62 ± 1.01 of males = 0.41 ± 0.86 & females = 0.97 ± 1.44,
**4**	**(Khedekar et al., 2015)** [73]	6 – 11	Both: 50 males & 50 females	city of Pune- India	100	Data not available	Median: 0.5 Range: 0–4 Mean is reported for every year of age separately
**5**	**(Kong et al., 2017)** [72]	3–5, 12–15	Both: 278 males & 444 females	Jiangbei & Fulling Districts- Chongqing- China	**722** 3-5Y: 246 12-15Y: 476	Data not available	**3-5Y:** dmft = 3.91 ± 1.12, **12-15Y:** DMFT = 0.89 ± 1.19
**6**	**(Marasouli et al., 2016)** [40]	6–18 (6–12, 13–18)	Both: 70 males & 23 females	Urmia- Iran	**93**	Data not available	**Females:** dmft = 1.89 **Males:** dmft = 2.92 **6-12Y:** DMFT = 1.38 **13-18Y:** DMFT = 2.96
**7**	**(Mohan et al., 2014)** [77]	5–14	Both: 70 males & 90 females	Lucknow city- India	**160:** 80 **(O)** Vs 80 **(C)**	1 Gov. Vs 1 NGO vs 1 private	Caries prevalence in **(O)** 83.7% vs 51.2% in **(C)**
**8**	**(Shah et al., 2016)** [70]	4–13 (4–6, 7–11, > 12)	Both: 964 males & 411 females	Jammu & Kashmir	**1,375**	Gov., NGO & private	**deft** (**4–6 years**) 1.355 ± 1.79 (**7–11 years**) 1.03 ± 1.61 **DMF**: **7-11yrs** 1.56 ± 1.85 ** > 12 yrs** 1.74 ± 1.92
**9**	**(Shanthi et al., 2017)** [78]	5, 12, 15	Both: 116 males & 137 females	Selangor- Malaysia	**253**	Data not available	Caries prevalence = 44.6%
**10**	**(Shuangjiao et al., 2014)** [69]	4–17 (4–12, 13–17)	Both: 179 males & 138 females	Jiangbei District-Chongqing City- China	**317**	Data not available	dmft = 1.94 ± 2.81 DMF = 0.90 ± 1.38
**11**	**(Suresan et al., 2021)** [71]	3–18	Both: 389 males & 340 females	Bhubaneswar city- Odisha- India	**729**	Gov.& aided	dmft = **0.96 ± 2.11** DMFT = **1.07 ± 1.56**
**12**	**(Thetakala et al., 2017)** [45]	6–15	Both: 474 males & 483 females	Mysore city- India	**957:** 478 **(O)** vs 479 **(C)**	Data not available	**defs = **2.72 ± 4.4 **DMFS:** 1.72 ± 2.3

*C* Control group, *Gov* Governmental, *NGO* Non-governmental organization, *O* Orphanage group

All the included studies were cross-sectional in design. The majority of them were performed in India, followed by three studies in each of Iran and China; then a single study held in each of Yemen, Egypt, Lithuania, and Malaysia. The included studies mostly recruited participants of both genders; with the exception of a study that was restricted to males [12] and another that did not report the participants’ demographic data [67]. The nine studies answering the first question mostly included children at the age of the mixed dentition stage; while the rest twelve recruited participants of a wider age range. In all cases, information about the orphanage/s and living circumstances -in which the studies were held- were not always reported.

The included studies reported a group of caries determinants: age, gender, oral hygiene status, oral hygiene practices, multimedia habits, sugar consumption, snacking, salivary buffer capacity, salivary bacterial content, intelligence quotient, self-concept, residence and type of orphanage. The types of caries determinants assessed in each of the included studies were highlighted in Table 3.

**Table 3:**
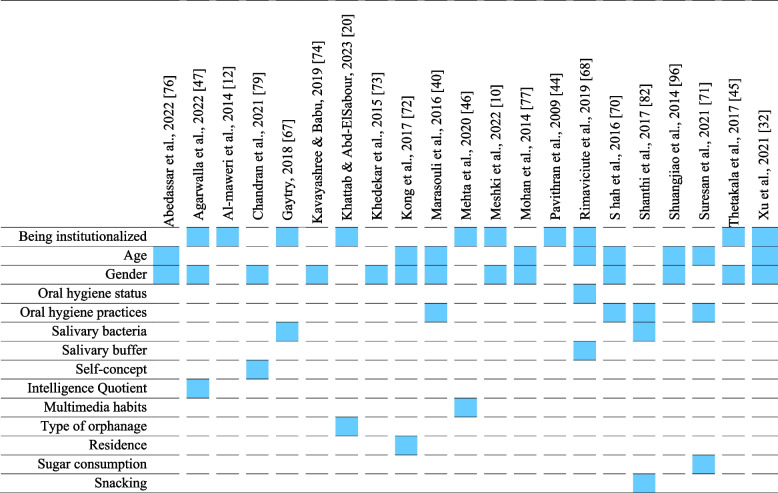
The risk factors assessed in all included studies Abedassar et al., 2022 [76], Agarwalla et al., 2022 [47], Al‑maweri et al., 2014 [12], Chandran et al., 2021 [79], Gaytry, 2018 [67], Kavayashree & Babu, 2019 [74], Khattab & Abd-ElSabour, 2023 [20], Khedekar et al., 2015 [73], Kong et al., 2017 [72], Marasouli et al., 2016 [40], Mehta et al., 2020 [46], Meshki et al., 2022 [10], Mohan et al., 2014 [77], Pavithran et al., 2009 [44], Rimaviciute et al., 2019 [68], Shah et al., 2016 [70], Shanthi et al., 2017 [78], Shuangjiao et al., 2014 [69], Suresan et al., 2021 [71], Thetakala et al., 2017 [45], Xu et al., 2021 [32]

### Risk of bias in studies

The reviewers assessed the risk of bias of the included studies using AHRQ (Additional file 3). As all the included studies were cross-sectional, no follow up was applicable in any of them. Therefore, the last domain of AHRQ (follow up) was omitted in assessing the included studies. Among the ten domains according to which studies were assessed, all studies proved low risk of bias regarding the source of data, reporting eligibility criteria and quality of the assessment method. Masking of the outcome assessor of the child’s condition was not applicable in most of the included studies; therefore, it was considered of low risk in these studies. However, three included studies assessed more than one outcome where each was reported by a different assessor [46, 47, 67]. In these studies, none reported if the assessors were blinded to the other outcome; therefore, were judged as having an unclear risk of bias.

**Fig. 2:**
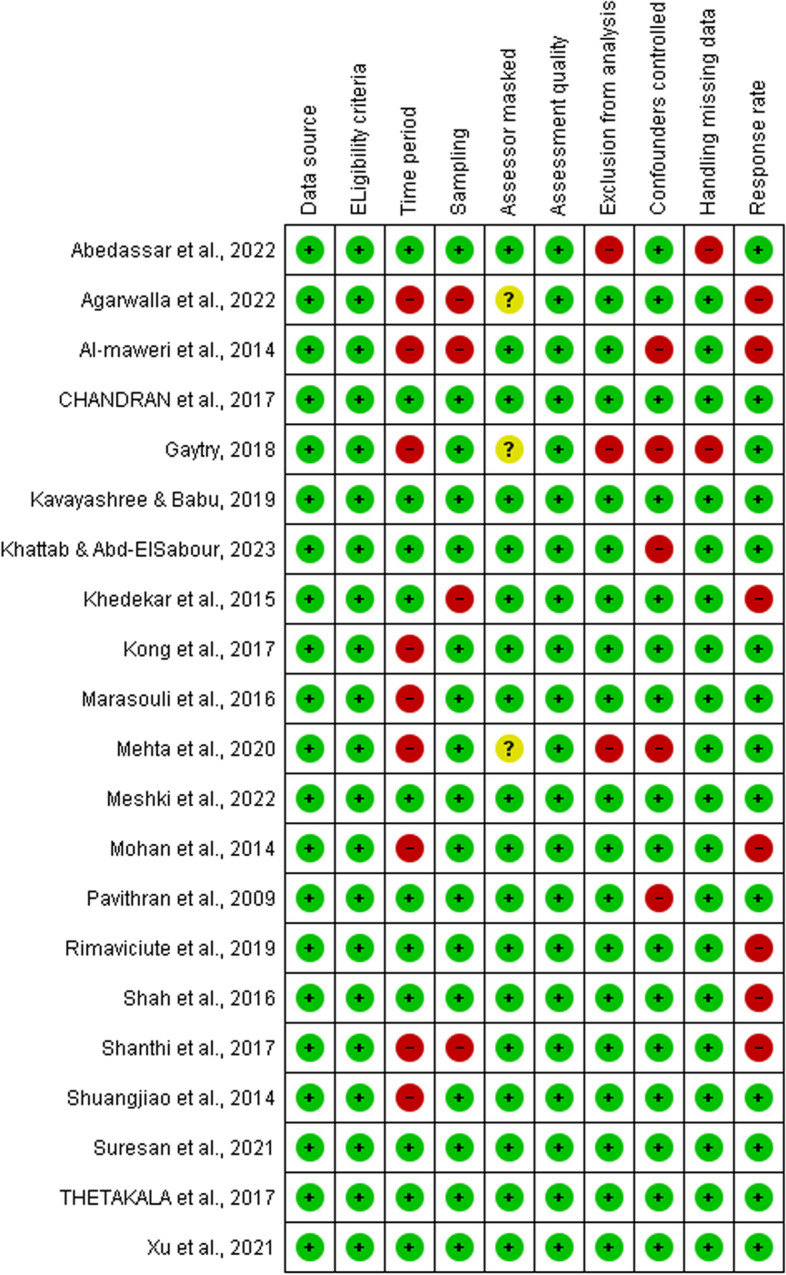
Risk of bias assessment of the included studies

The highest domains reported to have high risk of bias were reporting the response rate and reporting the time period in which the study was performed. Response rate was considered of high risk when the study did not report the sample size calculation, but instead sampled all the population of a certain orphanage; yet did not report the number of individuals who declined participation and the cause of their declining. Less number of studies were judged to have high risk of bias regarding controlling confounders, sampling, excluding participants from analysis and handling missing data (Fig. 2).

Judging individual studies, six out of the included 21 studies were assessed to have low risk of bias in all domains [10, 32, 45, 71, 74, 75].

### Results of individual studies

*Caries experience in institutionalized children Vs parented ones*: The results of caries experience in permanent teeth differed greatly between studies where some reported higher indices in institutionalized children when compared to their parented counterparts [10, 12, 20, 32, 44, 68]; contrasted by others [46, 47, 67]. Besides, most of the studies presented statistically insignificant results. Similar difference in results was observed regarding caries in primary teeth.

#### Caries determinants in institutionalized children

Some dental caries determinants have been tested in the included studies. In the following section, the determinants are ranked in order of the most commonly tested:

##### Age

Dental assessment is classified into 3 age categories depending on type of dentition: (a) Fully primary dentition (Less than 6 years), (b) Mixed dentition (6-up to 12 years) and (c) Fully permanent dentition (from 12-18 years).

A group of the included studies compared between the caries experience of some age groups:

- *Compared the primary to fully permanent:* Caries in primary teeth during the fully primary dentition was reported to be significantly higher than that in permanent teeth in the fully permanent dentition stage [32, 69–72].
- *Compared the mixed with the permanent dentition:* caries experience was observed to be higher in the age of (13–18) when compared to mixed dentition stage [40, 68, 76].
- *Compared the 3 stages together*: [69–71]

##### Gender

Thirteen of the included studies compared the results of their male and female participants [10, 32, 40, 45, 47, 69, 70, 72–74, 76, 77, 79]. However, the studies showed heterogeneous results; where some reported higher caries levels in males [10, 40], contrasted by others favoring females [74, 75], and some detected no significant difference [32, 45, 69, 70, 72, 73, 76, 77].

Furthermore, a single study [47] reported the results of all participants -orphans and parented- as one group. Therefore, the effect of gender difference on institutionalized orphans was not possibly concluded.

##### Oral hygiene practices

Oral hygiene practices were assessed using a questionnaire about the method of teeth cleaning, materials used for cleaning, and the frequency of brushing [40, 70, 71, 78]. From the mentioned oral hygiene practices, only vertical tooth brushing was statistically associated with the presence of dental caries [71].

Another study [70] reported high prevalence of caries among users of datum sticks, those who reported never using an oral hygiene method and those who never replaced their toothbrushes.

These results are corroborated by other studies [40, 78] where individuals brushing their teeth once were reported to have higher caries indices when compared to those brushing twice. On the other hand, no statistically significant difference was noticed between caries experience of individuals brushing twice and those brushing three times per day.

Other studies [12, 20, 73, 79] included oral hygiene practices as an outcome but did not correlate its results to the results of caries experience.

##### Oral hygiene status

One study [68] assessed oral hygiene through the Silness and Loe plaque index. The results showed a significant correlation between oral hygiene indices and the caries experience of the participants.

Other studies [10, 71, 73, 74] included oral hygiene status as a separate outcome but did not correlate its results to the caries experience.

##### Salivary bacteria concentration

Two of the included studies detected the levels of Streptococci and Lactobacilli in saliva [67, 78]. Unfortunately, the first study [78] did not correlate the bacterial concentration to caries detected.

On the other hand, the other study [67] tested whole stimulated saliva by Gram staining and Catalase tests after agar plating. However, the caries experience results of the institutionalized group were mostly skewed towards children having low caries scores (DMF= 1-3); while a small percentage of the group had higher DMF scores. Consequently, bacteria with all its gradient concentrations were detected in the mild-caries group more than the high-caries group. Of course, statistically sound inferences correlating the concentration of bacteria to the extent of caries are not plausible to be produced with a small number of participants in one subgroup versus a large number in another.

##### Salivary buffering effect

Salivary buffer capacity was found to be significantly positively correlated with caries levels in institutionalized children [68]**.**

##### Self-concept

Self-concept is the term describing the way an individual perceives oneself. It is subdivided into self-satisfaction about multiple domains: physical, social, temperamental, educational, moral, and intellectual. A strong negative correlation was reported between caries experience at one side and physical, social, temperamental, moral and intellectual dimension of self-concept of institutionalized orphans (p=0.0001) [75]. It is suggested that low levels of self-concept may have a psychological impact that would provoke oral problems through hygiene neglect.

##### Intelligence Quotient

Intelligent quotient (IQ) describes the relative intelligence of an individual expressed in the form of a score. A single study [47] correlated IQ scores with caries experience in institutionalized and parented children. The study reported institutionalized children to have significantly higher levels (23.02 ±1.84) of IQ scores than parented children (21.76 ±3.34). Concomitantly, lower levels of DMF scores were reported in institutionalized children (1.54 ±2.09) when compared with parented children (2.52 ±3.04). However, when correlating the level of IQ with caries experience, the results of the 2 groups were pooled together. The results showed the highest caries levels in children with (below average IQ), followed by children having (above average IQ); while the lowest caries levels were reported at children having (average IQ). But, the effect of IQ on institutionalized children was not reported solely in this study.

##### Multimedia habits

One study [46] was concerned with the habits related to time spent on following multimedia and the snacks eaten during this time. The study included institutionalized and parented children. However, the authors only reported the results of the parented children; with no simple statement of the results of the institutionalized group. They just mentioned that the habits of the whole group of institutionalized children were similar; owing to their limited access to multimedia and to advertised food. That being said, no association was possible between the habits and caries intensity in institutionalized children. So, unfortunately, the effect of multimedia following on institutionalized children can not be concluded from this study.

##### Type of orphanage

Three included studies [20, 70, 77] recruited residents from the 3 types of organizations: governmental, non-governmental organizations (NGOs) and private orphanages. Besides, a fourth study [71] included participants from governmental and NGO orphanages.

Despite that, only one study [20] performed subgroup analysis of the caries experience based on the sampling institute. Instead, the rest of the studies pooled the results of all the participants into one group.

The study [20] reported the highest def in NGO residents followed by parented children then governmental orphanages residents. On the other hand, the highest DMF was detected in NGO residents followed by governmental orphanages residents then parented children. The high caries indices in NGO residents were attributed to the poor oral hygiene and improper dietary habits; while the lower indices in governmental orphanage residents were believed to be due to their restricted diet.

##### Residence

Both dmf of orphans aged 3-5 years and DMF of orphans aged 12-15 years were significantly higher in rural orphanages when compared to residents of urban ones [72]**.** The authors attributed this difference to the more professional training that the staff receives in the city more than that in rural areas together with the higher oral health awareness levels.

##### Sugar consumption and snacking

Sugar consumption habits were assessed regarding frequency (number of times/day) and time of intake (within or in-between meals), form (solid or liquid), and consistency (sticky or not). The higher frequency of sugar intake was the only variable that showed significant association with the presence of dental caries [71]. Likewise, another study [78] reported that the caries prevalence was found to be significantly higher with more number of sweet snacks.

### Results of syntheses

**Fig. 3:**
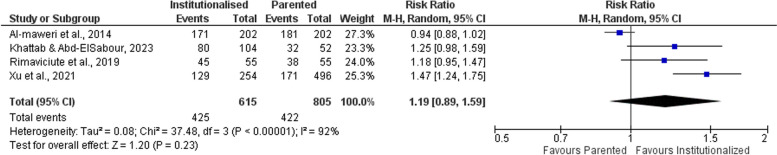
Forest plot showing risk ratio of caries in institutionalized children compared to parented ones

1. Caries experience in institutionalized children Vs parented ones:

First, the included studies were combined to detect the effect (risk ratio) of being institutionalized on caries prevalence. Caries prevalence was only reported by 4 of the included studies [12, 20, 32, 68]. The pooled estimate showed higher risk of caries in institutionalized children when compared to their parented counterparts (1.19 [0.89, 1.59]). The combined results showed that institutionalized children have 1.19 times the risk of having dental caries compared to those living with their families (Fig. 3).

Afterwards, the extent of caries experience (DMF/dmf) was compared between the 2 groups. Two records were not included in this analysis [67, 68] as the caries experience was not reported in the form of mean and standard deviation. In one study [67], the DMF scores were dichotomized and the caries experience was reported as the number of participants having more than 3 and less than 3. In the other study [68], the DMF of the control group was reported in the form of median unlike the orphanage group that was expressed as mean.

A solitary study [20] included 3 groups of children: group of orphans in an NGO institution, another group in a governmental institution and a control group. During analysis of the results, the authors dichotomized this study into 2 comparisons: one comparing NGO residents to controls and another comparing governmental institution residents to controls.

**Fig. 4:**
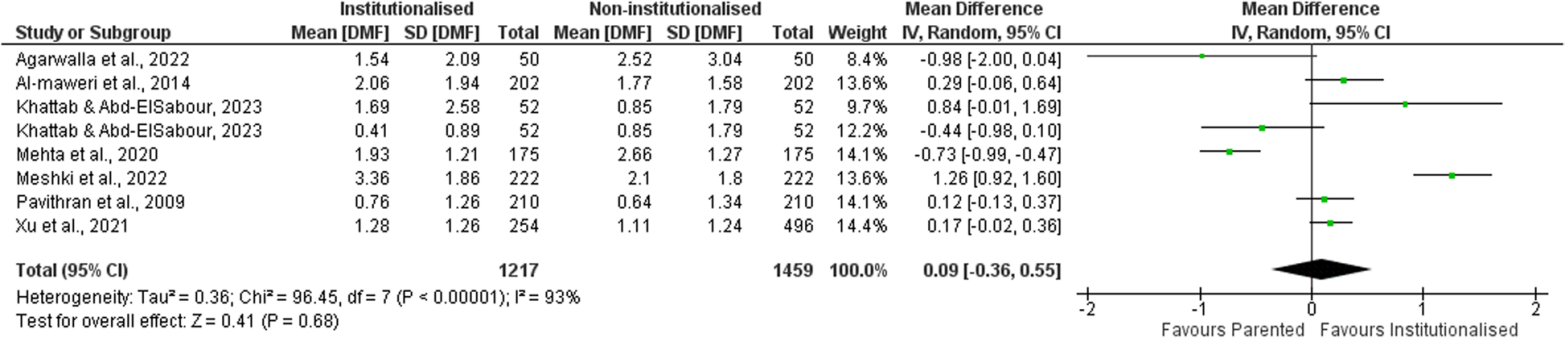
Forest plot showing the mean difference of caries experience in permanent teeth (DMF) between institutionalized and parented children

As the DMF results among the included studies were heterogeneous, combining such results yielded a statistically insignificant pooled estimate of (0.09) with its confidence interval crossing the point of no difference [-0.36, 0.55] (Fig. 4).

**Fig. 5:**
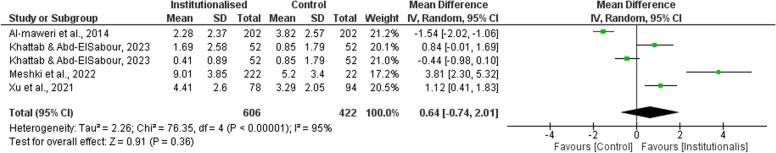
Forest plot showing the mean difference of caries experience in primary teeth (dmf) between institutionalized and parented children

Lastly, the caries experience of participants regarding the primary teeth (dmf) was combined among the four studies reporting them [10, 12, 20, 32]. Pooling of the results showed statistically insignificant higher caries experience in institutionalized children (0.64 [-0.74, 2.01]) (Fig. 5).

2. Caries determinants in institutionalized orphan children:

#### Age (according to the type of dentition)

**Fig. 6:**
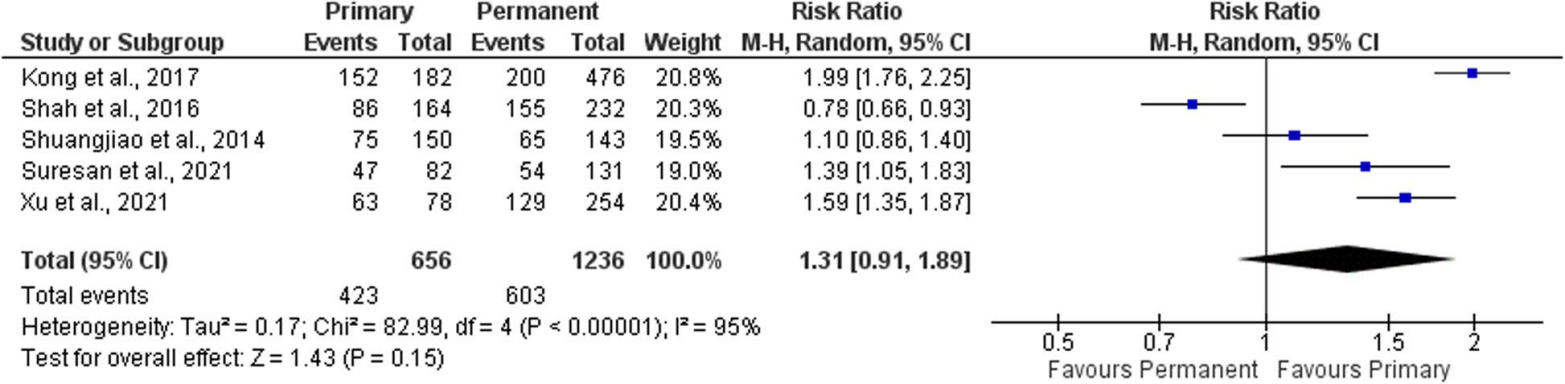
Forest plot showing risk ratio of caries in primary versus permanent teeth in institutionalized children

When comparing the caries experience in fully primary to fully permanent dentition ages, meta-analysis suggests that primary teeth have 1.31 times the risk of caries compared to permanent teeth. (relative risk=1.31 [0.91, 1.89]) (Fig. 6).

#### Gender

The method of reporting caries varied in the included studies between caries percentage and DMF. Therefore, only the results of the 5 studies that reported the caries prevalence (percentage) were possible to include in meta-analysis to conclude the effect of gender as a risk factor [69, 70, 72, 75, 77].

**Fig. 7:**
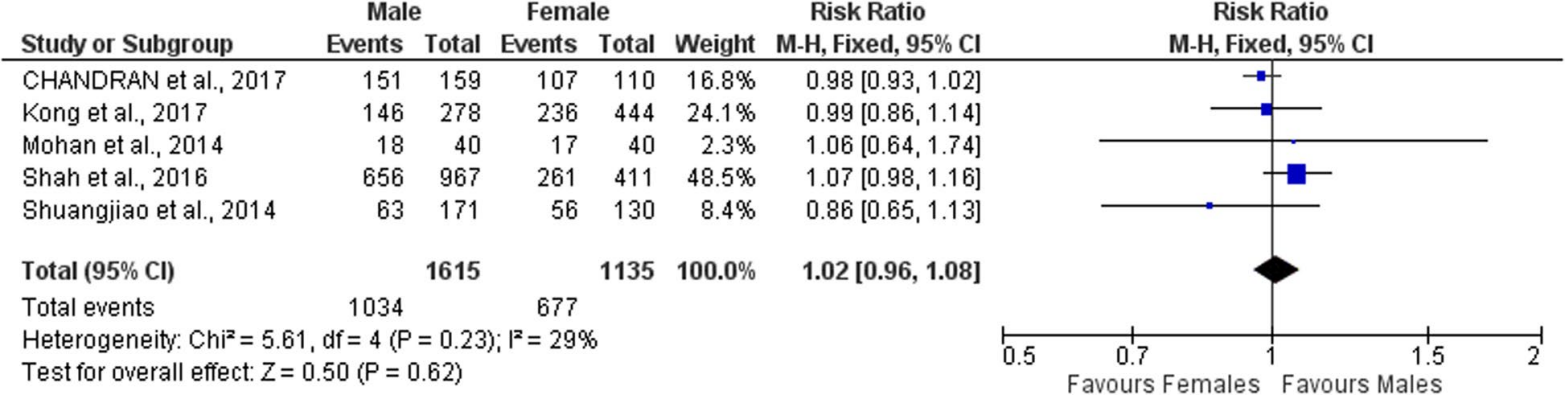
Forest plot showing risk ratio of caries in males versus females in institutionalized children

Fortunately, the studies involved in the meta-analysis include the ones having the highest number of participants among all the included studies in the review and so, the highest weight. The results showed slight difference between the 2 genders; with males being more vulnerable (1.02 [0.96, 1.08]) (Fig. 7).

#### Reporting biases

In each of the analyses, the included studies did not exceed ten in number. Therefore, testing the funnel plot symmetry would not be feasible due to low power of the test.

### Certainty of evidence

In all the outcomes of the review, all the eligible studies were observational, were limited in number, had considerable risk of bias, had heterogeneous inconsistent results, and mostly had low sample size. Therefore, the results concluded in this review are graded as being of very low quality of evidence.

## Discussion

Institutionalized children represent a considerable percentage of the world population, with a reported poor living conditions affecting their lives in almost all aspects. Regarding oral health, dental caries is proven to be the most prevalent disease in the world. Consequently, its debilitating effects on the individual’s well-being and quality of life is well established in literature [3, 80].

Therefore, a tremendous number of studies was concerned with investigating the prevalence of dental caries among institutionalized orphans. However, with the presence of this huge amount of data gathered from all around the world along a whole century’s time, no previous review addressed this disease in such marginalized populations; especially with the contradicting results of the individual reports. Therefore, this review was performed aiming to reflect the competitive state of dental caries among institutionalized children, in comparison to their parented counterparts; in addition to highlighting the determinants that affect the dental caries status in institutionalized children.

Determining the prevalence of such debilitating disease among such socially handicapped population is expected to provide a scientific background, according to which attempts to improve the dental health status of those children, and consequently their overall health condition, can be achieved [81].

The review adopted a systematic search where all relevant reports were reviewed. However, the finally included studies were conducted only in seven countries: India [44–47, 67, 70, 71, 73, 74, 77, 79], Iran [10, 40, 76], China [32, 69, 72], Yemen [12], Egypt [20], Lithuania [68], and Malaysia [78]. This can be explained through the strict eligibility criteria adopted by the review; where a huge number of studies were excluded for including medically compromised children (as clarified in Additional file 2). For the review to conclude reliable results, compromising medical conditions of the participants had to be excluded as they act as a major confounder to dental caries occurrence [78]. With the aforementioned eligibility criteria, the included studies were observed to be conducted in developing countries. Therefore, the final results of the review reflect the economic standards of the countries in which the studies were held.

Regarding the primary outcome of the review, the pooled caries score of permanent teeth (DMF) was found to be higher in institutionalized children when compared to that in parented controls (mean difference = 0.09 [-0.36, 0.55]). Similarly, pooling of the results of studies reporting caries experience of primary teeth (dmf score) showed higher caries experience in institutionalized children than that of their parented counterparts (0.64 [-0.74, 2.01]). However, in both meta-analyses, the results were statistically insignificant.

These results were clarified through the results of the meta-analysis estimating the effect of institutionalization on caries risk. It was shown that institutionalization increases the risk of having dental caries by 19% (RR = 1.19 [0.89, 1.59]).

The higher caries risk and poorer caries experience -revealed in the meta-analyses figures- may be attributed to the absence of parental influence on the institutionalized children, lack of proper parental supervision especially regarding oral hygiene measures and dietary control, deficient financial support, and insufficient professional dental follow-ups for these children. All the above-mentioned factors were reported to generally influence child’s caries experience and are known to be deficient in the institutionalized children’s groups [6, 7, 82].

As for the determinants, caries among institutionalized children is modulated by an array of determinants. Some factors were reported by multiple studies; making meta-analysis possible to estimate these factors’ effect on caries development in institutionalized orphans. The included studies highlighted the higher risk of caries in primary teeth more than permanent ones; and also the increasing caries risk in permanent teeth with increasing age. Furthermore, a slightly higher risk of caries was concluded for male gender.

Concerning age, permanent teeth of institutionalized orphans showed lower risk of caries compared to primary teeth. This finding may be owing to the change in dietary habits regarding the higher consumption of cariogenic snacks among children at a young age, in comparison to those in older age [83]. In addition to the lack of proper supervision on orphan children at young age; while in older ages, the child can manage to perform oral hygiene measures in a better way without supervision [84].

It was also observed that the caries experience in permanent teeth in children aged from 13 to 18 years was higher than that of children aged from 6 to 12 years. This could be attributed to the fact that the permanent teeth in the mixed dentition stage are newly erupted teeth and had not been exposed to cariogenic factors for sufficient time to develop dental caries [85]. Also, the number of permanent teeth in the mixed dentition stage is less than in that the fully permanent dentition stage; which makes the probability of children in the fully permanent dentition stage to have a higher mean total DMF score increased [85].

On the other hand, males were found to be at a higher risk of developing dental caries. Their risk was shown to be slightly higher -by 2%- when compared to females. This finding goes in line with what was reported in the general children population; where males are described to be at a higher risk of caries when compared to females. The lower risk in females was attributed to the nature of females being more keen about their oral hygiene and self-image than males [7].

Moreover, other risk factors were reported, each by an individual study; where the evidence of its results depends on the study quality and reporting. These studies reported a higher prevalence of caries in institutionalized orphans having the following risk factors: rural residence, low levels of self-concept, low salivary buffer capacity, less strict food discipline, consuming sugar with high frequency especially in snacks, high plaque index, and never using oral hygiene measures or brushing only once. All these factors were also proven to negatively influence caries experience among the general children population [8, 86, 87]. As in the general population, rural residents suffer from insufficient professional dental care, in addition to improper knowledge about oral health and hygiene measures [88]. Lack of self-concept, which is the image that the children have about themselves, was also proved to be a risk factor for developing dental caries in children [89].

While the authors aimed to specify the determinants of dental caries in institutionalized orphans, most of the included studies barely tested the effect of general determinants affecting the general pediatric population. Determinants that are specific to the studied population were not reported; including: number of years spent in the orphanage, age at the time of joining the orphanage, ruling methods of the orphanage, caregiver/orphan ratio, type and frequency of dental care provided, and funding source of the orphanage.

Generally, the pooled results of most of the review outcomes yielded statistically insignificant estimates. This can be attributed to the variation in the direction of effect between the individual studies. As an example, when comparing the caries experience of permanent teeth between orphans and parented children, some studies reported higher caries scores among the orphan children group while others reported the contrary. This difference may be due to differences between studies regarding baseline demographic and geographic data of the participants, social and financial levels of the included families and institutes, or difference in factors related to the institution's governance. Such factors influence the prevalence of dental caries among different institutions and families; and are -unfortunately- insufficiently reported in the included studies [7, 90, 91].

It is assumed that the difference in the available financial resources [6, 92], along with the availability of professional dental consultation [16], in addition to the knowledge, attitude, and practice of caregiver/ parents towards dental health [82, 93], all are attributing factors that govern the difference in the reported results among the included studies.

Besides, each of the included articles followed a separate protocol regarding the types of determinants measured, the methods of their measurement and the methods of their statistical analysis and reporting. The variation in the types of reported caries determinants among the included studies contributed to the present heterogeneity. All these factors were further added to the already known causes of clinical heterogeneity as the wide age range, the variation of the female to male ratio, and the difference in ethnicity among the included studies [94].

Therefore, this review was limited by the heterogeneity of the included studies, with inadequate and inconsistent reporting of the contributory factors that affect caries in each of the included studies. Besides, most of the included studies were of low-quality evidence. Nonetheless, the review highlights the need for professional dental healthcare for institutionalized children in orphanages, along with adequate oral health education for both children and caregivers.

Consequently, we suggest future studies should consider following a standard protocol in which caries experience should be assessed as both caries percentage and mean DMF/dmf, the numbers of participants should be reported at each step from recruitment to results analyses, caries determinants specific to institutionalized orphans should be included among the reported baseline data (number of years spent in the orphanage, age at the time of joining the orphanage, ruling methods of the orphanage, caregiver/orphan ratio, type and frequency of dental care provided, and funding source of the orphanage) and these factors should be correlated to the oral health results.

## Conclusions

Within the limitations of the current review, it could be concluded that institutionalized orphan children are at higher risk of developing dental caries, compared to their parented counterparts. Rural residence, low levels of self-concept, low salivary buffer capacity, less strict food discipline, consuming sugar with high frequency, especially in snacks, high plaque index, and never using oral hygiene measures or brushing only once, were all suggested as possible determining factors.

### Supplementary Information

**Additional file 1.** Search strategies for the two research outcomes.

**Additional file 2.** Table of excluded records with the justification of their exclusion.

**Additional file 3.** Risk of bias assessment checklists of all included studies.

## Data Availability

No datasets were generated or analysed during the current study.

## Abbreviations

AHRQ: Agency for Healthcare Research and Quality tool
C-OIDP: Child Oral Impacts on Daily Performances
def: Decayed-extracted-filled
DMF/ dmf: Decayed-missed-filled
IQ: Intelligence Quotient
NGOs: Non-governmental organizations
OHRQoL: Oral health-related quality of life
