# Supplementary material for: Caries in orphan children: prevalence and determinants—a systematic review and meta-analysis
Source: BMC Oral Health. 2024 Mar 25;24:381. doi: 10.1186/s12903-024-04125-9 (PMC10964678; doi:10.1186/s12903-024-04125-9)
Supplement: Supplementary file 1 — Additional file 1. Search strategies for the two research outcomes. [file 12903_2024_4125_MOESM1_ESM.docx]

**Search strategies:**

Question 1:

**Search Strategy**• **Search on PubMed (Draft)*:**

1. Orphan
2. Institutionalized
3. Institution
4. Orphanage

5. (1) OR (2) OR (3) OR (4)

6. child

7. children

1. pediatric
2. "Child"[Mesh]
3. (6) OR (7) OR (8) OR (9)
4. Parented
5. Family
6. (11) OR (12)
7. Tooth caries
8. tooth decay
9. Dental caries
10. Caries, dental [MeSH Terms]
11. Nursing caries
12. Early childhood caries
13. (14) OR (15) OR (16) OR (17) OR (18) OR (19).
14. (5) AND (10) AND (13) AND (18).

*Each line in the search strategy includes search words and each line is numbered sequentially by a reference number to the exact search word used in it. When a line includes numbers linked with the Boolean expressions (AND) or (OR), this indicates that the contents of the lines carrying these numbers (the search words referred to by these numbers) are to be gathered by the Boolean expression; rather than the numbers itself.

Question 2:

• **Search on PubMed (Draft)*:**

1. Orphan

2. Institutionalized

3. Institution

4. Orphanage

5. (1) OR (2) OR (3) OR (4)

6. child

7. children

8. pediatric

9. "Child"[Mesh]

10. (6) OR (7) OR (8) OR (9)

11. Tooth caries

12. Tooth decay

13. Dental caries

14. Caries, dental [MeSH Terms]

15. Nursing Caries

16. Early childhood caries

17. (11) OR (12) OR (13) OR (14) OR (15) OR (16).

18. (5) AND (10) AND (17).

*Each line in the search strategy includes search words and each line is numbered sequentially by a reference number to the exact search word used in it. When a line includes numbers linked with the Boolean expressions (AND) or (OR), this indicates that the contents of the lines carrying these numbers (the search words referred to by these numbers) are to be gathered by the Boolean expression; rather than the numbers itself.
