# Supplementary material for: Caries in orphan children: prevalence and determinants—a systematic review and meta-analysis
Source: BMC Oral Health. 2024 Mar 25;24:381. doi: 10.1186/s12903-024-04125-9 (PMC10964678; doi:10.1186/s12903-024-04125-9)
Supplement: Supplementary file 2 — Additional file 2. Table of excluded records with the justification of their exclusion. [file 12903_2024_4125_MOESM2_ESM.docx]

**Excluded records (Question 1):**

|  | **Record** | **Cause of Exclusion** |
| --- | --- | --- |
|  | (Abdul Majeed & Al-Dahan, 2017) | Outcomes did not include caries assessment |
|  | (Alsaif et al., 2022) | No exclusion of medically compromised individuals  excluded the participants with physical and mental condition. |
|  | (Amaral et al., 2021) | Included medically compromised participants  Ineligible caries experience assessment method |
|  | (Baghdadi, 2011) | Review article |
|  | (BUNTING et al., 1930) | No exclusion of medically compromised individuals |
|  | (El-Jobair et al., 2013) | Included medically compromised participants |
|  | (Gaur et al., 2014) | No exclusion of medically compromised individuals |
|  | (Kar et al., 2015) | Ineligible caries experience assessment method |
|  | (Karishma A. et al., 2020) | No exclusion of medically compromised individuals  Ineligible caries experience assessment method |
|  | (Kaur et al., 2017) | Outcomes did not include caries assessment |
|  | (Kumar et al., 2011) | No exclusion of medically compromised individuals |
|  | (Macphee, 1933) | Review article of irrelevant subject (paleodentistry) |
|  | (Maro & Kahabuka, 2007) | Ineligible caries experience assessment method  No exclusion of medically compromised individuals |
|  | (Masood et al., 2019) | Outcomes did not include caries assessment  Participants did not include orphans |
|  | (Mathur & Dhillon, 2018) | Review article |
|  | (Matulaitien, 2020) | Outcomes did not include caries assessment |
|  | (Miller & Crombie, 1939) | Participants did not include orphans |
|  | (Mohan et al., 2014) | Ineligible caries experience assessment method |
|  | (MORIOKA et al., 1964) | No exclusion of medically compromised individuals |
|  | (Narepekha & Dubetska-Harbous, 2016) | No exclusion of medically compromised individuals |
|  | (Navarro et al., 2012) | Review article |
|  | (Sinha et al., 2017) | No exclusion of medically compromised individuals |
|  | (Știrb & Petruș-Vancea, 2020) | Ineligible caries experience assessment method  No exclusion of medically compromised individuals |
|  | (THETAKALA et al., 2017) | Ineligible caries experience assessment method |

**Excluded records (Q2):**

|  | **Record** | **Cause of Exclusion** |
| --- | --- | --- |
|  | (Abdul Majeed & Al-Dahan, 2017) | Outcomes did not include caries assessment |
|  | (Ahlawat et al., 2019) | Outcomes did not include caries assessment  Included medically compromised participants |
|  | (Al-Malik & Holt, 2000) | No determinant measured |
|  | (Alsaif et al., 2022) | No exclusion of medically compromised individuals |
|  | (Alsayeh et al., 2020) | No exclusion of medically compromised individuals |
|  | (Amaral et al., 2021) | Included medically compromised participants  Ineligible caries experience assessment method |
|  | (Ayebameru et al., 2019) | No exclusion of medically compromised individuals |
|  | (Babu & Kavyashree, 2021) | Double publication with  (Kavayashree & Babu, 2019) |
|  | (Baghdadi, 2011) | Review article |
|  | (Bánóczy et al., 1985) | No exclusion of medically compromised individuals |
|  | (Bennadi et al., 2018) | No exclusion of medically compromised individuals |
|  | (Boyd & Cheyne, 1947a) | No exclusion of medically compromised individuals  Age range is not eligible  Baseline results are not reported |
|  | (Boyd & Cheyne, 1947b) | No exclusion of medically compromised individuals  Age range is not eligible  Baseline results are not reported |
|  | (Brewer et al., 1970) | Included medically compromised participants (mentally retarded) |
|  | (BUNTING et al., 1930) | No exclusion of medically compromised individuals |
|  | (Bunting et al., 1931) | No exclusion of medically compromised individuals |
|  | (Camacho et al., 2009) | No exclusion of medically compromised individuals |
|  | (Castellanos, 1974) | No exclusion of medically compromised individuals |
|  | (Christian et al., 2019) | No exclusion of medically compromised individuals |
|  | (Creighton & Wells, 1966) | No exclusion of medically compromised individuals |
|  | (Dmitrova & Kulakov, 2012) | Included medically compromised participants |
|  | (El-Jobair et al., 2013) | Included medically compromised participants |
|  | (Gandhi et al., 2018) | No exclusion of medically compromised individuals  Only physically and mentally handicapped |
|  | (Gaur et al., 2014) | No exclusion of medically compromised individuals |
|  | (Guimarães, 2017) | Included medically compromised participants (psychiatric disorders and on psychiatric medications) |
|  | (Gunawardane et al., 2015) | No exclusion of medically compromised individuals |
|  | (Goyal et al., 2011) | Double publication to (Kumar et al., 2011) |
|  | (HADJIMARKOS & STORVICK, 1949) | No exclusion of medically compromised individuals |
|  | (Hans et al., 2014) | No exclusion of medically compromised individuals  Ineligible age range (till 19) |
|  | (Hawkins, 1931) | No exclusion of medically compromised individuals |
|  | (Kahabuka & Mbawalla, 2006) | No exclusion of medically compromised individuals |
|  | (Kamran et al., 2017) | No exclusion of medically compromised individuals |
|  | (Kar et al., 2015) | No determinant measured |
|  | (Karishma A. et al., 2020) | No exclusion of medically compromised individuals |
|  | (Kaur et al., 2017) | Outcomes did not include caries assessment |
|  | (Khare et al., 2012) | No determinant measured |
|  | (Koong et al., 2013) | No exclusion of medically compromised individuals |
|  | (KOROLENKOVA et al., 2022) | No exclusion of medically compromised individuals |
|  | (KOYUNCUOĞLU et al., 2017) | No exclusion of medically compromised individuals |
|  | (Kulakov & Dmitrova, 2013) | Included medically compromised participants |
|  | (Kumar et al., 2011) | No exclusion of medically compromised individuals |
|  | (Macphee, 1933) | Review article of irrelevant subject (paleodentistry) |
|  | (Mahanta et al., 2022) | No exclusion of medically compromised individuals  Ineligible age range |
|  | (Manac’h, 2018) | Participants were not all orphans nor were they all institutionalized, but families supported by NGO |
|  | (Maro & Kahabuka, 2007) | No exclusion of medically compromised individuals |
|  | (Masood et al., 2019) | Outcomes did not include caries assessment  Participants did not include orphans |
|  | (Mathur & Dhillon, 2018) | Review article |
|  | (Matulaitien, 2020) | Outcomes did not include caries assessment |
|  | (Mazhari et al., 2008) | No exclusion of medically compromised individuals |
|  | (Miller & Crombie, 1939) | Participants did not include orphans |
|  | (MORIOKA et al., 1964) | No exclusion of medically compromised individuals |
|  | (Muralidharan et al., 2012) | No exclusion of medically compromised individuals |
|  | (Narepekha & Dubetska-Harbous, 2016) | No exclusion of medically compromised individuals |
|  | (Navarro et al., 2012) | Review article |
|  | (NEWBRUN, 1989) | Included medically compromised participants (mentally retarded) |
|  | (Pakdaman & Aref, 2012) | No exclusion of medically compromised individuals  RCT |
|  | (Paradnikaitė, 2019) | No exclusion of medically compromised individuals |
|  | (Pentapati et al., 2014) | No exclusion of medically compromised individuals |
|  | (Pratap et al., 2016) | No determinant measured |
|  | (Rashid & Othman, 2022) | No exclusion of medically compromised individuals |
|  | (Rea, 2015) | No exclusion of medically compromised individuals  Ineligible age range |
|  | (Roberts et al., 1938) | No exclusion of medically compromised individuals  Interventional study |
|  | (Santhosh et al., 2008) | No exclusion of medically compromised individuals |
|  | (Scheinin, Bánóczy, et al., 1985) | No exclusion of medically compromised individuals (Only hearing and visual impairment were excluded) |
|  | (Scheinin, Pienihäkkinen, et al., 1985) | No exclusion of medically compromised individuals (Only hearing and visual impairment were excluded)  Longitudinal (follow up of the first article) |
|  | (SChreiber & Scales, 1971) | No exclusion of medically compromised individuals  Used caries experience as risk factor not outcome, no determinants measured |
|  | (Shanbhog et al., 2013) | No exclusion of medically compromised individuals |
|  | (Shanbhog et al., 2014) | No exclusion of medically compromised individuals |
|  | (A. Sharma et al., 2014) | No exclusion of medically compromised individuals |
|  | (S. Sharma et al., 2019) | No exclusion of medically compromised individuals |
|  | (D. K. Singh et al., 2021) | No exclusion of medically compromised individuals |
|  | (R. K. Singh et al., 2022) | No exclusion of medically compromised individuals |
|  | (Sinha et al., 2017) | No exclusion of medically compromised individuals |
|  | (Solis-Riggioni et al., 2018) | No exclusion of medically compromised individuals |
|  | (Soni et al., 2020) | No exclusion of medically compromised individuals |
|  | (Srinivas et al., 2012) | No exclusion of medically compromised individuals |
|  | (Știrb & Petruș-Vancea, 2020) | No exclusion of medically compromised individuals |
|  | (Stoica et al., 2022) | No determinant measured |
|  | (Sudhakar et al., 2010) | No exclusion of medically compromised individuals |
|  | (O’Sullivan & Stephens, 1997) | No exclusion of medically compromised individuals |
|  | (Virk et al., 2012) | No exclusion of medically compromised individuals |
|  | (Zusman et al., 2014) | No exclusion of medically compromised individuals |

**References:**

Abdul Majeed, A. M., & Al-Dahan, Z. A. A. (2017). Oral Health Status in Relation to Nutritional Status among Institutionalized and Non-Institutionalized Orphans in Baghdad City. *Journal of Baghdad College of Dentistry*, *29*(4), 102–109. https://doi.org/10.12816/0043005

Ahlawat, P., Vyas, S., Sharma, N., Srivastava, A., Srivastava, A., & Semwal, J. (2019). Can personal hygiene act as a proxy indicator of morbidity profile? A study on orphans of Uttarakhand region. *International Journal Of Community Medicine And Public Health*, *6*(9), 3835. https://doi.org/10.18203/2394-6040.ijcmph20193980

Al-Malik, M., & Holt, R. D. (2000). The prevalence of caries and of tooth tissue loss in a group of children living in a social welfare institute in Jeddah, Saudi Arabia. *International Dental Journal*, *50*(5), 289–292. https://doi.org/10.1111/j.1875-595x.2000.tb00568.x

Alsaif, A. A., Alkhadra, T. A., & AlJameel, A. B. H. (2022). Impact of DMFT, PUFA, DAI, and TDIs on Oral Health-Related Quality of Life (OHRQoL) Among Foundling, Delinquent, and Mainstream School Children: A Prilimenary Study. *Frontiers in Public Health*, *10*(July), 1–8. https://doi.org/10.3389/fpubh.2022.894638

Alsayeh, A., Abdulbaseer, M., Aljelaly, H., Alshamrani, B., Aldaijy, R., Alshlash, K., Odeh, T., & Alshiha, A. (2020). Dentition Status and Treatment Needs Among Orphans in Riyadh City , Saudi Arabia : A Cross-sectional Descriptive Study Abstract : *The Open Dentistry Journal*, *14*, 203–210. https://doi.org/10.2174/1874210602014010203

Amaral, C. O. F. Do, Nascimento, C. T. Do, Granjeiro, L. D. S., Amaral, M. O. F. Do, & Straioto, F. G. (2021). Assessment of children oral health quality in institutional care situation Design of Study. *Australian Journal of Basic and Applied Sciences*, *15*(10), 20–26. https://doi.org/10.22587/ajbas.2021.15.10.3.ABSTRACT

Ayebameru, O. E., Popoola, B., & Denloye, O. (2019). Prevalence and predictors of dental caries and trauma among institutionalised and non-institutionalised street children in Ibadan. *African Journal of Oral Health*, *9*(1), 21–35. https://doi.org/10.4314/ajoh.v9i1.4

Babu, K. L. G., & Kavyashree, G. H. (2021). Evaluation of Oral Health ‑ Related Quality of Life among Institutionalized Orphan Children. *Journal of Forensic Scince and Medicine*, *7*, 117–122. https://doi.org/10.4103/jfsm.jfsm

Baghdadi, Z. D. (2011). Managing dental caries in children in Saudi Arabia. *International Dental Journal*, *61*(2), 101–108. https://doi.org/10.1111/j.1875-595X.2011.00021.x

Bánóczy, J., Scheinin, A., Esztári, I., Szöke, J., Hadas, E., & Zimmermann, P. (1985). Xilittartalmú édességek cariespreventív hatásának vizsgálata gyermekotthonokban [Caries-preventing action of xylitol-containing sweets, studied in children’s institutions]. *Orv Hetil*, *Oct 6 126*(40), 2447–2451.

Bennadi, D., Shabanam, S., Abdul, Nn., Jacob, A., Malini, K., & Bharateesh, J. (2018). Oral health status of orphanage children, Tumkur: A survey report. *International Journal of Community Dentistry*, *6*(2), 27. https://doi.org/10.4103/ijcd.ijcd_3_18

Boyd, J. D., & Cheyne, V. D. (1947a). Epidemiologic Studies in Dental Caries. II. The Incidence of Caries Among Institutionalized Children. *J Pediatrics*, *31*, 306–321.

Boyd, J. D., & Cheyne, V. D. (1947b). Epidemiologic studies in dental caries. *The Journal of Pediatrics*, *31*(3), 306–321. https://doi.org/10.1016/s0022-3476(47)80243-4

Brewer, H. E., Stookey, G. K., & Muhler, J. C. (1970). A clinical study concerning the anticariogenic effects of NaH2PO4-enriched breakfast cereals in institutionalized subjects: results after two years. *Journal of the American Dental Association (1939)*, *80*(1), 121–124. https://doi.org/10.14219/jada.archive.1970.0029

BUNTING, R. W., HADLEY., F. P., JAY, P., & HARD, D. G. (1930). The problem of dental caries. *American Journal of Diseases of Children*, *March*.

Bunting, R. W., Jay, P., & Hard, D. G. (1931). A Report of the Successful Control of Dental Caries in Three Public Institutions. *The Journal of the American Dental Association*, *18*(4), 672–678. https://doi.org/10.14219/jada.archive.1931.0124

Camacho, G. A., Camacho, E., Rodríguez, R. A., Guillé, A. J., Juárez, H. M., & Pérez, M. G. (2009). Predisposing factors for dental caries in girls at an orphanageof Mexico City. *Acta Pediatr Mex*, *30*(2), 71–76.

Castellanos, R. A. (1974). [Epidemiological aspects of dental caries in white and non-white schoolchildren of both sexes admitted to seven orphanages in the city of São Paulo, Brazil, in 1972] Aspectos epidemiológicos da cárie dental em escolares brancos e não brancos de ambos os s. *Revista de Saúde Pública*, *8*(1), 51–62. https://doi.org/10.1590/s0034-89101974000100006

Christian, B., Ummer-christian, R., Blinkhorn, A., Hegde, V., Nandakumar, K., Marino, R., & Chattopadhyay, A. (2019). An epidemiological study of dental caries and associated factors among children residing in orphanages in Kerala , India : Health in Orphanages Project ( HOPe ). *International Dental Journal*, *69*(2), 113–118. https://doi.org/10.1111/idj.12419

Creighton, W. E., & Wells, H. B. (1966). Dental Caries Experience in Institutionalized Mongoloid and Nonmongoloid Children in North Carolina and Oregon. *Journal of Dental Research*, *45*(1), 66–75. https://doi.org/10.1177/00220345660450013301

Dmitrova, A. G., & Kulakov, A. A. (2012). [Dental caries in young and primary school age children in institutions for orphans] Kariyes zubov u detey doshkol’nogo i mladshego shkol’nogo vozrasta v detskikh domakh. *Stomatologiia (Mosk)*, *1*, 62–64.

El-Jobair, A. M., Al-Sadham, S. A., Al-faifi, A. A., Andijani, R. I., & Al-Motlag, S. K. (2013). Medical and dental health status of orphan children in central Saudi Arabia. *Saudi Med J*, *34*(5), 531–536.

Gandhi, S. R., Gandhi, R., & Lecturer, S. (2018). ASSESSMENT OF ORAL HEALTH STATUS AND TREATMENT NEEDS OF CHILDREN RESIDING IN ORPHANAGE HOME IN CENTRAL INDIA-A CROSS SECTIONAL STUDY. *Journal of Interdisciplinary Dental Sciences*, *7*(1), 31–35.

Gaur, A., Sujan, S. G., & Katna, V. (2014). The oral health status of institutionalized children that is , Juvenile home and orphanage home run by Gujarat state Government , in Vadodara city with that of normal school children. *Journal of Indian Society of Pedodontics and Preventive Dentistry*, *32*(3), 231–237. https://doi.org/10.4103/0970-4388.135833

Goyal, A., Sharma, A., Singh, S., & Patel, R. (2011). Oral Health Related Quality of Life among Children with Parents and those with No Parents. *JOURNAL OF THE INDIAN ASSOCIATION OF PUBLIC HEALTH DENTISTRY*, *18 Suppl 1*, 501–507.

Guimarães, A. C. S. S. (2017). *Characterization of Oral Health in Institutionalized Children and Young People in the District of Porto [Caracterização da Saúde Oral em Crianças e Jovens Institucionalizados no Distrito do Porto]*.

Gunawardane, S. R., Angammana, H. M. R. W., Palanage, N. N., Bannaheka, B. M. H. S. K., Fonseka, M. C. N., & Bandara, J. M. J. (2015). *Oral Health Status and Treatment Needs Among Institutionalized Children In The Central Province of Sri Lanka*. *45*(03), 85–93.

HADJIMARKOS, D. M., & STORVICK, C. A. (1949). GEOGRAPHIC VARIATIONS OF DENTAL CARIES IN OREGON II. DENTAL CARIES AMONG INSTITUTIONALIZED CHILDREN AND THE POSSIBLE INFLUENCE OF CERTAIN ECOLOGICAL FACTORS ON ITS INCIDENCE. *J D ResD Res*, *28*(6), 594–599.

Hans, R., Thomas, S., Dagli, R., Bhateja, G. A., Sharma, A., & Singh, A. (2014). Oral health knowledge, Attitude and practices of children and adolescents of orphanages in jodhpur city rajasthan, India. *Journal of Clinical and Diagnostic Research*, *8*(10), ZC22–ZC25. https://doi.org/10.7860/JCDR/2014/9026.4948

Hawkins, H. F. (1931). A rational technique for the control of caries and systemic pyorrhea. *Journal of Dental Research*, *11*(2), 257–274.

Kahabuka, F., & Mbawalla, H. (2006). Oral health knowledge and practices among Dar es Salaam institutionalized former street children aged 7-16 years. *Int J Dent Hygiene*, *4*, 174–178.

Kamran, R., Farooq, W., Faisal, M. R., & Jahangir, F. (2017). Clinical consequences of untreated dental caries assessed using PUFA index and its covariates in children residing in orphanages of Pakistan. *BMC Oral Health*, *17*(1), 1–7. https://doi.org/10.1186/s12903-017-0399-9

Kar, S., Kundu, G., Ghosh, C., Jana, A., Kundu, D. K., & Maiti, S. K. (2015). A COMPARATIVE EVALUATION OF CARIES PREVALENCE AMONG ORPHAN AND NORMAL CHILDREN OF MALDA, WEST BENGAL EVALUATED WITH CARIES ASSESSMENT SPECTRUM AND TREATMENT-A RECENT CARIES ASSESSMENT SYSTEM. *World Journal of Pharmaceutical Research*, *4*(9), 2133–2138.

Karishma A., Anuj Nair, Anjali D., & Indrajeet Singh. (2020). Oral Health Status of Institutionalized Orphans and Non-Orphans Aged 6-12 Years in a Central Indian City: A Comparative Study. *International Healthcare Research Journal*, *4*(9), OR12–OR15. https://doi.org/10.26440/ihrj/0409.12360

Kaur, R., Singh, K., Puri, M. S., Anandani, C., & Pal Singh, H. (2017). Plaques Scores and Gingival Health Status between 5 To 20 Years Old Orphans and Parented Children. *Scholars Journal of Applied Medical Sciences*, *5*(6E), 2427–2431. https://doi.org/10.36347/sjams.2017.v05i06.069

Kavayashree, G., & Babu, K. L. G. (2019). Assessment of Oral Health Status of Children Living in Orphanages of Hassan City , India. *Journal of Indian Association of Public Health Dentostry*, *17*, 201–205. https://doi.org/10.4103/jiaphd.jiaphd

Khare, V., Koshy, A., Rani, P. J., Srilatha, S., Kapse, S. C., & Agrawal, A. (2012). Prevalence of dental caries and treatment needs among the orphan children and adolescents of Udaipur District, Rajasthan, India. *Journal of Contemporary Dental Practice*, *13*(2), 182–187. https://doi.org/10.5005/jp-journals-10024-1118

Koong, H., Song, E., & Hwang, S. (2013). [Oral Health and Quality of Life of the Orphans in Dong-gu, Daejeon] daejeon dong-gu boyug-wonsaeng-ui gugang-geongang mich gugang-geongang-gwanlyeon salm-ui jil- Korean. *J Dent Hyg Sci Vol.*, *13*(3), 223–229.

KOROLENKOVA, M. V., KHACHATRYAN, A. G., POBEREZHNAYA, A. A., & KRECHETOVA, M. S. (2022). [Dental caries prevention program in children and adolescents living in residential institutions] Model’ profilaktiki kariyesa zubov u detey i podrostkov, prozhivayushchikh v organizovannom detskom kollektive. *Stomatology*, *101*(4), 61–67.

KOYUNCUOĞLU, C. Z., KAZAK, M., PAMUK, F., & ÇİFCİBAŞI, E. (2017). ORAL HYGIENE HABITS AND ORAL HEALTH STATUS OF FEMALE ADOLESCENTS UNDER STATE PROTECTION: A PILOT STUDY. In *J Istanbul Univ Fac Dent* (Vol. 51, Issue 1, pp. 1–7).

Kulakov, A. A., & Dmitrova, A. G. (2013). [Various toothpastes efficiency in children living in orphan institutions]. Issledovaniye effektivnosti razlichnykh vidov zubnykh past v detskikh internatakh chl. *Stomatologii︠a︡a*, *92*(4), 62–65.

Kumar, S., Goyal, A., Tadakamadla, J., Tibdewal, H., Duraiswamy, P., & Kulkarni, S. (2011). Oral health related quality of life among children with parents and those with no parents. *Community Denral Health*, *28*, 227–231. https://doi.org/10.1922/CDH

Macphee, G. G. (1933). *D e n t a l Caries*.

Mahanta, P., Das Thakuria, K., Goswami, P., Kalita, C., Knower, R., Rajbangshi, M. C., Singh, S. G., Basumatary, J., & Majumder, P. (2022). Evaluation of physical and mental health status of orphan children living in orphanages in Sonitpur district of Assam: a cross-sectional study. *BMC Pediatrics*, *22*(1), 1–7. https://doi.org/10.1186/s12887-022-03785-2

Manac’h, J. J.-M. M. (2018). *Relationship between dental caries and BMI among NGO-PSE’s children in Phnom Penh, Cambodia*. http://dx.doi.org/10.1186/s13662-017-1121-6%0Ahttps://doi.org/10.1007/s41980-018-0101-2%0Ahttps://doi.org/10.1016/j.cnsns.2018.04.019%0Ahttps://doi.org/10.1016/j.cam.2017.10.014%0Ahttp://dx.doi.org/10.1016/j.apm.2011.07.041%0Ahttp://arxiv.org/abs/1502.020

Maro, D., & Kahabuka, F. (2007). Prevalence of early childhood caries among 2-6 years old underpriviledged and privileged children inn Dar es Salaam. In *Tanz Dent J* (Vol. 14, Issue 2, pp. 54–60).

Masood, M., Mnatzaganian, G., & Baker, S. R. (2019). Inequalities in dental caries in children within the UK : Have there been changes over time? *Community Dent Oral Epidemiol*, *47*, 71–77. https://doi.org/10.1111/cdoe.12426

Mathur, V. P., & Dhillon, J. K. (2018). Dental Caries : A Disease Which Needs Attention. *Indian Journal of Pediatrics*, *85*(3), 202–206. https://doi.org/10.1007/s12098-017-2381-6

Matulaitien, K. (2020). *Vaikų gyvenančių šeimose ir vaikų globos namuose burnos priežiūros būklė ir žinio s [CHILDREN FROM ORPHANAGES AND CHILDREN LIVING IN FAMILIES ORAL HEALTH STATUS AND KNOWLEDGE]*.

Mazhari, F., Ajami, B., & Ojrati, N. (2008). [Dental Treatment Needs of 6-12-year old Children in Mashhad Orphanages in 2006] Naazhaa dndanpezshkea kewdkean 6 ta 12 salh perwrshguah haa mshhd dr sal 1385. *Journal of Mashhad Dental School*, *32*(1), 81–86.

Miller, H., & Crombie, D. (1939). COMPLETE FREEDOM FROM DENTAL CARIES A COMPARATIVE STUDY OF TWENTY-FIVE CHILDREN BY. *The Lancet*, *July*(July), 131–133.

Mohan, A., Misra, N., Umapathy, D., Kumar, S., Srivastav, D., & Mohan, U. (2014). Oral and dental health status in orphan children of Lucknow. *Indian Journal of Community Health*, *26*(2), 170–173.

MORIOKA, T., HAMADA, K., UEDA, M., INOUE, S., & OSAKA, T. (1964). [Observation on dental caries experience and dietary analysis of the children of orphanages] Fuku Shi Yō-ji no kōkū shinsa to Sakae Yō chōsa yui hate nitsu ite- Japanese. *Journal of Oral Hygiene Society*, *14*(3), 1–14.

Muralidharan, D., Fareed, N., & Shanthi, M. (2012). Comprehensive dental health care program at an orphanage in Nellore district of Andhra Pradesh. *Indian J Dent Res*, *23*, 171–175.

Narepekha, O. T., & Dubetska-Harbous, I. S. (2016). [THE STATE OF HARD TISSUE OF TEMPORARY TEETH IN CHILDREN FROM ORPHANAGES AND BOARDING-SCHOOLS] STAN TVERDYKH TKANYN TYMCHASNYKH ZUBIV U DITEY LʹVIVSʹKYKH INTERNATIV. *Ukrainian Dental Almanac*, *3*(1), 100–103.

Navarro, M. F. de L., Modena, K. C. da S., & Bresciani, E. (2012). Social disparity and oral health. *Braz Oral Res*, *26*(Spec Iss 1), 17–24.

NEWBRUN, E. (1989). Frequent sugar intake – then and now: interpretation of the main results. *European Journal of Oral Sciences*, *97*(2), 103–109. https://doi.org/10.1111/j.1600-0722.1989.tb01437.x

O’Sullivan, E. A., & Stephens, A. J. (1997). The oral and dental status of children residing in a Romanian orphanage. *International Journal of Paediatric Dentistry*, *7*(1), 41–42. https://doi.org/10.1111/j.1365-263x.1997.tb00273.x

Pakdaman, A., & Aref, P. (2012). Evaluation of an oral health promotion program designed for pre-school children in Ameneh orphan center. *Journal of Dental Medicine-Tehran University of Medical Sciences*, *25*(1), 56–61.

Paradnikaitė, G. (2019). *Evaluation of Institutionalized Children’s Oral Health Care State and Estimation of Dental Services Accessibility*.

Pentapati, K., Yeturi, S., & Acharya, S. (2014). Unmet restorative treatment needs among orphanage children of Uttara Kannada District. *Journal of Education and Ethics in Dentistry*, *4*(2), 65. https://doi.org/10.4103/0974-7761.148989

Pratap, R., Puranik, M., & Uma, S. (2016). Caries experience and its relationship with oral health related quality of life among orphanage children in Bengaluru City: A cross-sectional study. *Journal of Indian Association of Public Health Dentistry*, *14*(4), 397. https://doi.org/10.4103/2319-5932.195834

Rashid, B., & Othman, Z. Y. (2022). Initial Impact of an Educational Program Programme for the Orphans in Erbil City. *Eurasian Journal of Science and Engineering*, *8*(1), 180–186. https://doi.org/10.23918/eajse.v8i1p180

Rea, M. (2015). Haitian orphan p opulation and protective factors against c aries. *Dentistry 3000*, *3*(1), 1–5. https://doi.org/10.5195/d3000.

Roberts, L., Englebrecht, S., Blair, R., Williams, W., & Scott, M. (1938). Effect of a milk supplement on the physical status of institutional children. III. Progress of dental caries. *American Journal of Disease*, *56*(4), 805–823.

Santhosh, K., Jyothi, A., Prabu, D., & Suhas, K. (2008). Oral hygiene and periodontal status among children and adolescents residing at an orphanage in Udaipur city , India. *Nig Dent J*, *16*(2), 82–86.

Scheinin, A., Bánóczy, J., Szöke, J., Esztári, I., Pienihäkkinen, K., Scheinin, U., Tiekso, J., Zimmermann, P., & Hadas, E. (1985). Collaborative WHO xylitol Field studies in Hungary I. Three-year caries activity in institutionalized children. *Acta Odontologica Scandinavica*, *43*(6), 327–347. https://doi.org/10.3109/00016358509046517

Scheinin, A., Pienihäkkinen, K., Tiekso, J., Bánóczy, J., Szöke, J., Esztári, I., Zimmermann, P., & Hadas, E. (1985). Collaborative WHO xylitol Field studies in Hungary VII. Two-year caries incidence in 976 institutionalized children. *Acta Odontologica Scandinavica*, *43*(6), 381–387. https://doi.org/10.3109/00016358509046523

SChreiber, E. H., & Scales, J. L. (1971). Anxiety and dental health in institutionalized delinquent adolescent. *Journal of the American Dental Association (1939)*, *82*(3), 600–602. https://doi.org/10.14219/jada.archive.1971.0097

Shanbhog, R., Godhi, B. S., Nandlal, B., Kumar, S. S., Raju, V., & Rashmi, S. (2013). Clinical consequences of untreated dental caries evaluated using PUFA index in orphanage children from India. *Journal of International Oral Health : JIOH*, *5*(5), 1–9. http://www.ncbi.nlm.nih.gov/pubmed/24324297%0Ahttp://www.pubmedcentral.nih.gov/articlerender.fcgi?artid=PMC3845277

Shanbhog, R., Raju, V., & Nandlal, B. (2014). Correlation of oral health status of socially handicapped children with their oral heath knowledge, attitude, and practices from India. *Journal of Natural Science, Biology and Medicine*, *5*(1), 101–107. https://doi.org/10.4103/0976-9668.127297

Sharma, A., Gaur, A., Pareek, S., Raja, V., Sanadhya, S., & Sharma, A. B. (2014). Oral Health Status and Treatment Needs among Orphanage Children of Jaipur City. *Scholars Journal of Applied Medical Sciences (SJAMS*, *2*(5D), 1776–1780. https://doi.org/10.36347/sjams.2014.v02i05.058

Sharma, S., Shahi, A. K., Chandra, S., Kumari, M., Singh, R. N. P., & Singh, B. (2019). Dental caries and periodontal status among 6-15 yrs aged orphanage children in Patna, Bihar: A cross sectional study. *J Adv Med Dent Scie Res*, *7*(10), 107–111. https://doi.org/10.21276/jamdsr

Singh, D. K., Gupta, A., Hameed, S., & Kumar, S. N. (2021). Health Status of Children Residing at Orphanages in Rural Karnataka. *Journal of Medical Sciences and Health*, *7*(2), 43–46. https://doi.org/10.46347/jmsh.2021.v07i02.008

Singh, R. K., Thakar, R. G., Simple Kaur, & Daisy Naidu. (2022). Assessment of Oral Health Status among Orphanage Children (Underprivileged Population): A Descriptive Cross-Sectional Study. *International Healthcare Research Journal*, *5*(10), OR1–OR4. https://doi.org/10.26440/ihrj/0510.01503

Sinha, A., Kaur, K., Singh, K., Puri, M. S., Anandani, C., & Kaur, J. (2017). DENTAL CARIES STATUS AMONG ORPHANS AND PARENTED CHILDREN IN NORTH INDIA: A COMPARATIVE STUDY. *J Adv Med Dent Scie Res*, *5*(6), 54–57. https://doi.org/10.21276/jamdsr.2017.5.6.14

Solis-Riggioni, A., Gallardo-Barquero, C., & Chavarria-Bolaños, D. (2018). Prevalence and severity of dental caries in foster-care children and adolescents. *Journal of Clinical Pediatric Dentistry*, *42*(4), 269–272. https://doi.org/10.17796/1053-4628-42.4.5

Soni, A., Sharma, H., Motghare, V., & Verma, S. (2020). Assessment of Oral Health Status in Orphanage Inmates of North ‑ Eastern Part of Rajasthan : A Descriptive Cross ‑ Sectional Study. *J Indian Association of Public Health Dentistry*, *18*, 139–142. https://doi.org/10.4103/jiaphd.jiaphd

Srinivas, R., Srinivas, P., Viswanath, V., Suresh, S., Devaki, T., & Narayana, V. (2012). Oral Health Status of Institutionalized Street Children Aged 5-15 Years In Guntur City , Andhra. *International Journal of Scientific Nad Technology Research*, *1*(11), 19–23.

Știrb, L., & Petruș-Vancea, A. (2020). RESEARCH CONCERNING THE OCCURRENCE OF DENTAL AFFECTIONS AT 11 - 12 YEARS OLD PUPILS , FROM ROMANIA RURAL AREA. *EDUCAȚIA OMULUI DE AZI PENTRU LUMEA DE MAINE*, *3*, 90–98.

Stoica, O. E., Esian, D., Bud, A., Stoica, A. M., Beresescu, L., & Bica, C. I. (2022). The Assessment of Early Server Childhood Caries Status in Abandoned Institutionalized Children. *International Journal of Environmental Research and Public Health*, *19*(14), 4–13. https://doi.org/10.3390/ijerph19148632

Sudhakar, K., RSharath, P., Shanthi, M., Fareed, N., & Sudhir, K. (2010). Relationship between Dentition status and Body Mass Index among 5 to 15 years old age group children of an orphanage in Nellore city. *Journal of Indian Association of Public Health Dentistry*, *8*(15), 45. https://doi.org/10.4103/2319-5932.197406

THETAKALA, R. K., SUNITHA, S., CHANDRASHEKAR, B., SHARMA, Pri., KRUPA, N., & SRILATHA, Y. (2017). Periodontal and Dentition Status among Orphans and Children with Parents in Mysore City , India : A Comparative Study. *Journal of Clinical and Diagnostic Research*, *11*(4), ZC115–ZC118. https://doi.org/10.7860/JCDR/2017/25655.9748

Virk, P. K. S., Jain, R. L., Pathak, A., Sharma, U., & Rajput, J. S. (2012). Inter-relationship of intelligence-quotient and self-concept with dental caries amongst socially handicapped orphan children. *Journal of Indian Society of Pedodontics and Preventive Dentistry*, *30*(2), 127–132. https://doi.org/10.4103/0970-4388.99986

Zusman, S. P., Eaton, K. A., Harris, M., & Amariei, C. (2014). A pilot project to improve the oral health of orphans and of the elderly in residential care in Constanta, Romania. *Community Dental Health*, *32*(2), 89–92. https://doi.org/10.1922/CDH_3435Zusman04
