# Supplementary material for: Caries in orphan children: prevalence and determinants—a systematic review and meta-analysis
Source: BMC Oral Health. 2024 Mar 25;24:381. doi: 10.1186/s12903-024-04125-9 (PMC10964678; doi:10.1186/s12903-024-04125-9)
Supplement: Supplementary file 3 — Additional file 3. Risk of bias assessment checklists of all included studies. [file 12903_2024_4125_MOESM3_ESM.docx]

**RoB assessment of all studies: Tools cited from** **(Zeng et al., 2015):**

1. **(Abedassar et al., 2022)**

AHRQ tool **for cross-sectional (yes/No/ Unclear)**

| **No.** | **Description of question** | **Criteria** | **Comment from the record (verbatim)** |
| --- | --- | --- | --- |
| **Q1** | Define the source of information  (Survey, record review) | **1= from survey**  2= not mentioned  3= records/ unclear info | “All managing personnel signed a written consent. Then, a short briefing was given to the children and all of them were included in the study voluntarily and knowingly.” |
| **Q2** | List inclusion and exclusion criteria for exposed and unexposed subjects (cases and controls) or refer to previous publications | **1= clearly mentioned**  2= no information  3= unclear / insufficient info | “Exclusion criteria of the study were undergoing dental treatment or having a predisposing systemic condition that affects dental and oral health.” |
| **Q3** | Indicate time period used for identifying subjects | **1= time period given**  2= no info given | “in the year 2019” |
| **Q4** | Indicate whether or not subjects were consecutive if not population based. | **1= representative**  2= not representative (convenience/ not randomly selected)  3= no clear info | “Kerman Province in southeast of Iran has the most boarding centers for children in the whole country and no study has covered this issue in this province.”  “**all** children aged between 6-18 years old living in 20 welfare boarding centers in Kerman City in the year 2019 and they were entered into the study by the census method.” |
| **Q5** | Indicate if evaluators of subjective components of study were masked to other aspects of the status of the participants. | 1= evaluator masked  **2= not masked**  3= unclear /not mentioned | **Comment:** Masking is not applicable  So **low risk of bias** |
| **Q6** | Describe any assessments undertaken for quality assurance purposes (e.g., test/retest of primary outcome measurements) | **1= exposure & outcome tools validated/examiner-kappa-score reported)**  2= not done  3= unclear /partially done | “then the calibrated dentist (kappa coefficient = 0.85) used…” |
| **Q7** | Explain any patient exclusions from analysis | 1=mentioned clearly  **2=not mentioned**  3=unclear information  4=NA | **Comment:** The study included 356 participants but the outcome was only measured in 341 participants. No mention of the cause of not including all. |
| **Q8** | Describe how confounding was assessed and/or controlled. | **1=mentioned (design/analysis)**  2=not done  3=unclear /not mentioned | **Comment:** Subgroup analysis of results regarding the age and gender |
| **Q9** | If applicable, explain how missing data were handled in the analysis | 1= clearly mentioned  **2= no information**  3= unclear / insufficient info |  |
| **Q10** | Summarize patient response rates and completeness of data collection | **1=mentioned & above 80%**  2=not mentioned  3=unclear information | “5 centers (including 75 people, 21.1%) did not share the information regarding the cause for the children to reside in the center and level of education of their parents.  The remaining 75 people (21%) did not answer and were not included in this data part.”  “two children, one of which had a cleft palate and the other was receiving orthodontic treatment, were removed from the study. In addition, one child refused to receive clinical examination after filling out the questionnaire and was excluded.”  **Comment:** yet the response is above 80% |
| **Q11** | Clarify what follow-up, if any, was expected and the percentage of patients for which incomplete data or follow-up was obtained | 1= clearly mentioned  2= not mentioned  **3= unclear / not applicable** | NA |

1. **(Agarwalla et al., 2022)**

| **No.** | **Description of question** | **Criteria** | **Comment from the record (verbatim)** |
| --- | --- | --- | --- |
| **Q1** | Define the source of information  (Survey, record review) | **1= from survey**  2= not mentioned  3= records/ unclear info |  |
| **Q2** | List inclusion and exclusion criteria for exposed and unexposed subjects (cases and controls) or refer to previous publications | **1= clearly mentioned**  2= no information  3= unclear / insufficient info | “The selection of children was carried out on the basis of the  following criteria:.” |
| **Q3** | Indicate time period used for identifying subjects | 1= time period given  **2= no info given** |  |
| **Q4** | Indicate whether or not subjects were consecutive if not population based. | 1= representative  **2= not representative (convenience/ not randomly selected)**  3= no clear info | “**All** inmates (both boys and girls) residing in the orphanage  during the study period.  • **All** school-going children living with their respective family members.”  **Comment:**  **NO** sample size calculation was reported.  **No** specification of the way of choosing the orphanages and schools. |
| **Q5** | Indicate if evaluators of subjective components of study were masked to other aspects of the status of the participants. | 1= evaluator masked  2= not masked  **3= unclear /not mentioned** | “clinical psychologist performed the proper scoring and  interpretation of the intelligence test. The dentist recorded dental  caries status of the participants.”  **Comment:** but no mention if the 2 assessors are masked to the other outcome results. |
| **Q6** | Describe any assessments undertaken for quality assurance purposes (e.g., test/retest of primary outcome measurements) | **1= exposure & outcome tools validated/examiner-kappa-score reported)**  2= not done  3= unclear /partially done | (RCPM) under the guidance of a clinical psychologist in each child aged 7 to 11 years. It is a most acceptable test for measuring cognitive abilities”  “Standardization of Investigator” |
| **Q7** | Explain any patient exclusions from analysis | 1=mentioned clearly  2=not mentioned  3=unclear information  **4=NA** | **Comment:** no patients excluded  So, low risk of bias |
| **Q8** | Describe how confounding was assessed and/or controlled. | **1=mentioned (design/analysis)**  2=not done  3=unclear /not mentioned | **Comment:** Subgroup analysis of results regarding the gender |
| **Q9** | If applicable, explain how missing data were handled in the analysis | 1= clearly mentioned  **2= no information**  3= unclear / insufficient info | **Comment**: no missing data; So, low risk of bias |
| **Q10** | Summarize patient response rates and completeness of data collection | 1=mentioned & above 80%  **2=not mentioned**  3=unclear information | **Comment**: As there is no sample size calculation and no specification of the capacity of the orphanages, the response rate can not be concluded. |
| **Q11** | Clarify what follow-up, if any, was expected and the percentage of patients for which incomplete data or follow-up was obtained | 1= clearly mentioned  2= not mentioned  **3= unclear / not applicable** | NA |

1. **(Al‑maweri et al., 2014)**

| **No.** | **Description of question** | **Criteria** | **Comment from the record (verbatim)** |
| --- | --- | --- | --- |
| **Q1** | Define the source of information  (Survey, record review) | **1= from survey**  2= not mentioned  3= records/ unclear info | “An interview questionnaire….” |
| **Q2** | List inclusion and exclusion criteria for exposed and unexposed subjects (cases and controls) or refer to previous publications | **1= clearly mentioned**  2= no information  3= unclear / insufficient info | “Children, in both groups, with any kind of disability or systemic disease were excluded from the study |
| **Q3** | Indicate time period used for identifying subjects | 1= time period given  **2= no info given** |  |
| **Q4** | Indicate whether or not subjects were consecutive if not population based. | 1= representative  **2= not representative (convenience/ not randomly selected)**  3= no clear info | “Controls were randomly  selected from one public school in the same neighborhood”  **Comment:** No statement of the sample size calculation and the method of selecting the orphanage group. |
| **Q5** | Indicate if evaluators of subjective components of study were masked to other aspects of the status of the participants. | 1= evaluator masked  **2= not masked**  3= unclear /not mentioned | **Comment:** Masking is not applicable  So **low risk of bias** |
| **Q6** | Describe any assessments undertaken for quality assurance purposes (e.g., test/retest of primary outcome measurements) | **1= exposure & outcome tools validated/examiner-kappa-score reported)**  2= not done  3= unclear /partially done | Dental status was evaluated using Decayed/decayed, Missed/missed and Filled/filled (DMFT/dmft) index according to the World Health Organization oral health surveys.[6] |
| **Q7** | Explain any patient exclusions from analysis | 1=mentioned clearly  2=not mentioned  3=unclear information  **4=NA** | **Comment:** No patients excluded  So, low risk of bias |
| **Q8** | Describe how confounding was assessed and/or controlled. | 1=mentioned (design/analysis)  **2=not done**  3=unclear /not mentioned |  |
| **Q9** | If applicable, explain how missing data were handled in the analysis | 1= clearly mentioned  **2= no information**  3= unclear / insufficient info | **Comment**: no missing data; So, low risk of bias |
| **Q10** | Summarize patient response rates and completeness of data collection | 1=mentioned & above 80%  **2=not mentioned**  3=unclear information | **Comment**: As there is no sample size calculation and no specification of the capacity of the orphanages, the response rate can not be concluded. |
| **Q11** | Clarify what follow-up, if any, was expected and the percentage of patients for which incomplete data or follow-up was obtained | 1= clearly mentioned  2= not mentioned  **3= unclear / not applicable** | NA |

1. **(CHANDRAN, 2017) (Chandran et al., 2021)**

AHRQ tool **for cross-sectional (yes/No/ Unclear)**

| **No.** | **Description of question** | **Criteria** | **Comment from the record (verbatim)** |
| --- | --- | --- | --- |
| **Q1** | Define the source of information  (Survey, record review) | **1= from survey**  2= not mentioned  3= records/ unclear info | “Data was collected using questionnaires and clinical examination was done. “ |
| **Q2** | List inclusion and exclusion criteria for exposed and unexposed subjects (cases and controls) or refer to previous publications | **1= clearly mentioned**  2= no information  3= unclear / insufficient info | ” Inmates with any handicapping conditions and systemic diseases or conditions were excluded from the study.” |
| **Q3** | Indicate time period used for identifying subjects | **1= time period given**  2= no info given | “from January 2016 to September 2016” |
| **Q4** | Indicate whether or not subjects were consecutive if not population based. | **1= representative**  2= not representative (convenience/ not randomly selected)  3= no clear info | “ Cluster sampling was done to obtain the participants. It consisted of 39 orphanages each of which served as a cluster…selected by lottery method and all members of the selected clusters were enrolled into the study.” |
| **Q5** | Indicate if evaluators of subjective components of study were masked to other aspects of the status of the participants. | 1= evaluator masked  **2= not masked**  3= unclear /not mentioned | **Comment:** Masking is not applicable  So **low risk of bias** |
| **Q6** | Describe any assessments undertaken for quality assurance purposes (e.g., test/retest of primary outcome measurements) | **1= exposure & outcome tools validated/examiner-kappa-score reported)**  2= not done  3= unclear /partially done | “A pilot study was conducted to find out the feasibility of the study, for training and calibration of the investigator and to check for the test–retest reliability of the questionnaire.” |
| **Q7** | Explain any patient exclusions from analysis | 1=mentioned clearly  2=not mentioned  3=unclear information  **4=NA** | **Comment:** No patients excluded; So, low risk of bias |
| **Q8** | Describe how confounding was assessed and/or controlled. | **1=mentioned (design/analysis)**  2=not done  3=unclear /not mentioned | **Comment:** Subgroup analysis of results regarding the gender |
| **Q9** | If applicable, explain how missing data were handled in the analysis | 1= clearly mentioned  **2= no information**  3= unclear / insufficient info | **Comment**: no missing data; So, low risk of bias |
| **Q10** | Summarize patient response rates and completeness of data collection | 1=mentioned & above 80%  **2=not mentioned**  3=unclear information | **Comment:** The sample size was fulfilled so low risk. |
| **Q11** | Clarify what follow-up, if any, was expected and the percentage of patients for which incomplete data or follow-up was obtained | 1= clearly mentioned  2= not mentioned  **3= unclear / not applicable** | Not applicable |

1. **(Gaytry, 2018)**

| **No.** | **Description of question** | **Criteria** | **Comment from the record (verbatim)** |
| --- | --- | --- | --- |
| **Q1** | Define the source of information  (Survey, record review) | **1= from survey**  2= not mentioned  3= records/ unclear info |  |
| **Q2** | List inclusion and exclusion criteria for exposed and unexposed subjects (cases and controls) or refer to previous publications | **1= clearly mentioned**  2= no information  3= unclear / insufficient info |  |
| **Q3** | Indicate time period used for identifying subjects | 1= time period given  **2= no info given** |  |
| **Q4** | Indicate whether or not subjects were consecutive if not population based. | **1= representative**  2= not representative (convenience/ not randomly selected)  3= no clear info | “The school going children were selected by two stage random sampling method” |
| **Q5** | Indicate if evaluators of subjective components of study were masked to other aspects of the status of the participants. | 1= evaluator masked  2= not masked  **3= unclear /not mentioned** | **Comment:** The saliva sample assessor could be blinded of the caries incidence of the child but no information is available in this regard. |
| **Q6** | Describe any assessments undertaken for quality assurance purposes (e.g., test/retest of primary outcome measurements) | **1= exposure & outcome tools validated/examiner-kappa-score reported)**  2= not done  3= unclear /partially done |  |
| **Q7** | Explain any patient exclusions from analysis | 1=mentioned clearly  **2=not mentioned**  3=unclear information  4=NA | **Comment:** testing of CFU in institutionalized group (table 5) was less than the total number by 2 with no justification. |
| **Q8** | Describe how confounding was assessed and/or controlled. | 1=mentioned (design/analysis)  **2=not done**  3=unclear /not mentioned |  |
| **Q9** | If applicable, explain how missing data were handled in the analysis | 1= clearly mentioned  **2= no information**  3= unclear / insufficient info | **Comment:** No data mentioned |
| **Q10** | Summarize patient response rates and completeness of data collection | 1=mentioned & above 80%  **2=not mentioned**  3=unclear information | **Comment:** The sample size was fulfilled so low risk. |
| **Q11** | Clarify what follow-up, if any, was expected and the percentage of patients for which incomplete data or follow-up was obtained | 1= clearly mentioned  2= not mentioned  **3= unclear / not applicable** | NA |

1. **(Kavayashree & Babu, 2019) and (Babu & Kavyashree, 2021)**

AHRQ tool **for cross-sectional (yes/No/ Unclear)**

| **No.** | **Description of question** | **Criteria** | **Comment from the record (verbatim)** |
| --- | --- | --- | --- |
| **Q1** | Define the source of information  (Survey, record review) | **1= from survey**  2= not mentioned  3= records/ unclear info | “normal and healthy  children aged 6–14 years participated by answering the itemized  questionnaire given to them.” |
| **Q2** | List inclusion and exclusion criteria for exposed and unexposed subjects (cases and controls) or refer to previous publications | **1= clearly mentioned**  2= no information  3= unclear / insufficient info | “Normal, healthy, cooperative orphan children, aged  6–14 years and residing in orphanages…………” |
| **Q3** | Indicate time period used for identifying subjects | **1= time period given**  2= no info given | over 6 months during January–June 2017. |
| **Q4** | Indicate whether or not subjects were consecutive if not population based. | **1= representative**  2= not representative (convenience/ not randomly selected)  3= no clear info | “The estimated sample size was 86 which was rounded off to 100.” |
| **Q5** | Indicate if evaluators of subjective components of study were masked to other aspects of the status of the participants. | 1= evaluator masked  **2= not masked**  3= unclear /not mentioned | **Comment:** Masking is not applicable  So **low risk of bias** |
| **Q6** | Describe any assessments undertaken for quality assurance purposes (e.g., test/retest of primary outcome measurements) | **1= exposure & outcome tools validated/examiner-kappa-score reported)**  2= not done  3= unclear /partially done | “Training and calibration for oral examination and diagnosis of  dental caries were carried…”  “Ten  percent of children were examined twice for intra‑examiner  reliability. The kappa value for the intra‑examiner agreement  was 0.88.” |
| **Q7** | Explain any patient exclusions from analysis | 1=mentioned clearly  2=not mentioned  3=unclear information  **4=NA** | **Comment:** No patients excluded; So, low risk of bias |
| **Q8** | Describe how confounding was assessed and/or controlled. | **1=mentioned (design/analysis)**  2=not done  3=unclear /not mentioned | **Comment:** Subgroup analysis of results regarding the gender |
| **Q9** | If applicable, explain how missing data were handled in the analysis | 1= clearly mentioned  **2= no information**  3= unclear / insufficient info | **Comment**: no missing data; So, low risk of bias |
| **Q10** | Summarize patient response rates and completeness of data collection | 1=mentioned & above 80%  **2=not mentioned**  3=unclear information | **Comment:** The sample size was fulfilled so low risk. |
| **Q11** | Clarify what follow-up, if any, was expected and the percentage of patients for which incomplete data or follow-up was obtained | 1= clearly mentioned  2= not mentioned  **3= unclear / not applicable** | Not applicable |

1. **(Khattab & Abd-ElSabour, 2023)**

| **No.** | **Description of question** | **Criteria** | **Comment from the record (verbatim)** |
| --- | --- | --- | --- |
| **Q1** | Define the source of information  (Survey, record review) | **1= from survey**  2= not mentioned  3= records/ unclear info | “All children were examined for….” |
| **Q2** | List inclusion and exclusion criteria for exposed and unexposed subjects (cases and controls) or refer to previous publications | **1= clearly mentioned**  2= no information  3= unclear / insufficient info | “*Inclusion*  *criteria* were cooperative, apparently…..” |
| **Q3** | Indicate time period used for identifying subjects | **1= time period given**  2= no info given | “during the last two weeks of November 2022.” |
| **Q4** | Indicate whether or not subjects were consecutive if not population based. | **1= representative**  2= not representative (convenience/ not randomly selected)  3= no clear info | “study sample was collected from three randomly selected settings”  “Sample size calculation was performed…” |
| **Q5** | Indicate if evaluators of subjective components of study were masked to other aspects of the status of the participants. | 1= evaluator masked  **2= not masked**  3= unclear /not mentioned | **Comment:** Masking is not applicable  So **low risk of bias**. |
| **Q6** | Describe any assessments undertaken for quality assurance purposes (e.g., test/retest of primary outcome measurements) | **1= exposure & outcome tools validated/examiner-kappa-score reported)**  2= not done  3= unclear /partially done | **“**inter-examiner and  intra-examiner calibration were established for both  investigators, …., and reliability was tested  and was proved to be within the accepted range (Kapa test = 0.89).” |
| **Q7** | Explain any patient exclusions from analysis | 1=mentioned clearly  2=not mentioned  3=unclear information  **4=NA** | **Comment:** No patients excluded; So, low risk of bias |
| **Q8** | Describe how confounding was assessed and/or controlled. | 1=mentioned (design/analysis)  **2=not done**  3=unclear /not mentioned |  |
| **Q9** | If applicable, explain how missing data were handled in the analysis | 1= clearly mentioned  **2= no information**  3= unclear / insufficient info | **Comment**: no missing data; So, low risk of bias |
| **Q10** | Summarize patient response rates and completeness of data collection | 1=mentioned & above 80%  **2=not mentioned**  3=unclear information | **Comment:** The sample size was fulfilled so low risk. |
| **Q11** | Clarify what follow-up, if any, was expected and the percentage of patients for which incomplete data or follow-up was obtained | 1= clearly mentioned  2= not mentioned  **3= unclear / not applicable** | NA |

1. **(Khedekar et al., 2015)**

AHRQ tool **for cross-sectional (yes/No/ Unclear)**

| **No.** | **Description of question** | **Criteria** | **Comment from the record (verbatim)** |
| --- | --- | --- | --- |
| **Q1** | Define the source of information  (Survey, record review) | **1= from survey**  2= not mentioned  3= records/ unclear info | “A specially designed questionnaire was answered by the orphans and…. “ “Intraoral examination was performed while the  child was in…” |
| **Q2** | List inclusion and exclusion criteria for exposed and unexposed subjects (cases and controls) or refer to previous publications | **1= clearly mentioned**  2= no information  3= unclear / insufficient info | ”Exclusion criteria were children suffering…..” |
| **Q3** | Indicate time period used for identifying subjects | **1= time period given**  2= no info given | “from April to June 2014” |
| **Q4** | Indicate whether or not subjects were consecutive if not population based. | 1= representative  **2= not representative (convenience/ not randomly selected**)  3= no clear info | **Comment:** No mentioning of the way of sampling or the sample size calculation |
| **Q5** | Indicate if evaluators of subjective components of study were masked to other aspects of the status of the participants. | 1= evaluator masked  **2= not masked**  3= unclear /not mentioned | **Comment:** Masking is not applicable  So **low risk of bias** |
| **Q6** | Describe any assessments undertaken for quality assurance purposes (e.g., test/retest of primary outcome measurements) | **1= exposure & outcome tools validated/examiner-kappa-score reported)**  2= not done  3= unclear /partially done | “Baseline data were collected on oral hygiene, according to WHO recommendations comprising DMFS (Decayed, Missing, Filled Tooth Surfaces Index, for permanent teeth), OHI-S (Simplified Oral Hygiene Index), and the Gingival Index” |
| **Q7** | Explain any patient exclusions from analysis | 1=mentioned clearly  2=not mentioned  3=unclear information  **4=NA** | **Comment:** No patients excluded; So, low risk of bias |
| **Q8** | Describe how confounding was assessed and/or controlled. | **1=mentioned (design/analysis)**  2=not done  3=unclear /not mentioned | **Comment:** Subgroup analysis of results regarding the age and gender |
| **Q9** | If applicable, explain how missing data were handled in the analysis | 1= clearly mentioned  **2= no information**  3= unclear / insufficient info | **Comment**: no missing data; So, low risk of bias |
| **Q10** | Summarize patient response rates and completeness of data collection | 1=mentioned & above 80%  **2=not mentioned**  3=unclear information | **Comment**: As there is no sample size calculation and no specification of the capacity of the orphanages, the response rate can not be concluded. |
| **Q11** | Clarify what follow-up, if any, was expected and the percentage of patients for which incomplete data or follow-up was obtained | 1= clearly mentioned  2= not mentioned  **3= unclear / not applicable** | Not applicable |

1. **(Kong et al., 2017)**

AHRQ tool **for cross-sectional (yes/No/ Unclear)**

| **No.** | **Description of question** | **Criteria** | **Comment from the record (verbatim)** |
| --- | --- | --- | --- |
| **Q1** | Define the source of information  (Survey, record review) | **1= from survey**  2= not mentioned  3= records/ unclear info | “All the oral examination were conducted by…” |
| **Q2** | List inclusion and exclusion criteria for exposed and unexposed subjects (cases and controls) or refer to previous publications | **1= clearly mentioned**  2= no information  3= unclear / insufficient info | ” Children who had major systemic diseases or who were on long-term medication were excluded from the  study…..” |
| **Q3** | Indicate time period used for identifying subjects | 1= time period given  **2= no info given** |  |
| **Q4** | Indicate whether or not subjects were consecutive if not population based. | **1= representative**  2= not representative (convenience/ not randomly selected)  3= no clear info | “ random sampling methods”  “With a standard error set at 1.5%, the sample size required in this survey would be…..” |
| **Q5** | Indicate if evaluators of subjective components of study were masked to other aspects of the status of the participants. | 1= evaluator masked  **2= not masked**  3= unclear /not mentioned | **Comment:** Masking is not applicable  So **low risk of bias** |
| **Q6** | Describe any assessments undertaken for quality assurance purposes (e.g., test/retest of primary outcome measurements) | **1= exposure & outcome tools validated/examiner-kappa-score reported)**  2= not done  3= unclear /partially done | ” Before  the survey, the standard conformance testing is completely reliable (Kappa values > 0.8).” |
| **Q7** | Explain any patient exclusions from analysis | 1=mentioned clearly  2=not mentioned  3=unclear information  **4=NA** | **Comment:** No patients excluded; So, low risk of bias |
| **Q8** | Describe how confounding was assessed and/or controlled. | **1=mentioned (design/analysis)**  2=not done  3=unclear /not mentioned | **Comment:** Subgroup analysis of results regarding the age, gender and residence. |
| **Q9** | If applicable, explain how missing data were handled in the analysis | 1= clearly mentioned  **2= no information**  3= unclear / insufficient info | **Comment**: no missing data; So, low risk of bias |
| **Q10** | Summarize patient response rates and completeness of data collection | 1=mentioned & above 80%  **2=not mentioned**  3=unclear information | **Comment:** The sample size was fulfilled so low risk. |
| **Q11** | Clarify what follow-up, if any, was expected and the percentage of patients for which incomplete data or follow-up was obtained | 1= clearly mentioned  2= not mentioned  **3= unclear / not applicable** | Not applicable |

1. **(Marasouli et al., 2016)**

AHRQ tool **for cross-sectional (yes/No/ Unclear)**

| **No.** | **Description of question** | **Criteria** | **Comment from the record (verbatim)** |
| --- | --- | --- | --- |
| **Q1** | Define the source of information  (Survey, record review) | **1= from survey**  2= not mentioned  3= records/ unclear info | “as well as their clinical examination, and questions…” |
| **Q2** | List inclusion and exclusion criteria for exposed and unexposed subjects (cases and controls) or refer to previous publications | **1= clearly mentioned**  2= no information  3= unclear / insufficient info | ” Finally, completely systematically healthy people were included, whose number reached 93 people, and one person who had a systemic problem was excluded…..” |
| **Q3** | Indicate time period used for identifying subjects | 1= time period given  **2= no info given** |  |
| **Q4** | Indicate whether or not subjects were consecutive if not population based. | **1= representative**  2= not representative (convenience/ not randomly selected)  3= no clear info | “All people between the ages of 6 and 18 years in Urmia care centers were included by census, which contained 94 people in 5 units.…..” |
| **Q5** | Indicate if evaluators of subjective components of study were masked to other aspects of the status of the participants. | 1= evaluator masked  **2= not masked**  3= unclear /not mentioned | **Comment:** Masking is not applicable  So **low risk of bias** |
| **Q6** | Describe any assessments undertaken for quality assurance purposes (e.g., test/retest of primary outcome measurements) | **1= exposure & outcome tools validated/examiner-kappa-score reported)**  2= not done  3= unclear /partially done | ” people were classified according to the results of the dmft and DMFT indicators, the method proposed by the World Health Organization.” |
| **Q7** | Explain any patient exclusions from analysis | **1=mentioned clearly**  2=not mentioned  3=unclear information  4=NA | **Quote**: “on 93 healthy children out of 96 individuals in 5 care centers in Urmia”  “All people between the ages of 6 and 18 years in Urmia care centers were included by census, which contained 94 people”  “Finally, healthy people were systematically included completely, whose number reached 93 people |
| **Q8** | Describe how confounding was assessed and/or controlled. | **1=mentioned (design/analysis)**  2=not done  3=unclear /not mentioned | **Comment:** Subgroup analysis of results regarding the age, gender and oral hygiene measures. |
| **Q9** | If applicable, explain how missing data were handled in the analysis | 1= clearly mentioned  **2= no information**  3= unclear / insufficient info | **Comment**: no missing data; So, low risk of bias |
| **Q10** | Summarize patient response rates and completeness of data collection | **1=mentioned & above 80%**  2=not mentioned  3=unclear information | **Quote**: “which contained 94 people”  “on 93 healthy children out of 96 individuals in 5 care centers in Urmia” |
| **Q11** | Clarify what follow-up, if any, was expected and the percentage of patients for which incomplete data or follow-up was obtained | 1= clearly mentioned  2= not mentioned  **3= unclear / not applicable** | Not applicable |

1. **(Mehta et al., 2020)**

| **No.** | **Description of question** | **Criteria** | **Comment from the record (verbatim)** |
| --- | --- | --- | --- |
| **Q1** | Define the source of information  (Survey, record review) | **1= from survey**  2= not mentioned  3= records/ unclear info | “Examination of the  participants was undertaken….” |
| **Q2** | List inclusion and exclusion criteria for exposed and unexposed subjects (cases and controls) or refer to previous publications | **1= clearly mentioned**  2= no information  3= unclear / insufficient info | “Children who are….. were excluded from the study. ” |
| **Q3** | Indicate time period used for identifying subjects | 1= time period given  **2= no info given** |  |
| **Q4** | Indicate whether or not subjects were consecutive if not population based. | **1= representative**  2= not representative (convenience/ not randomly selected)  3= no clear info | “Children belonging to a similar socioeconomic background from institutionalized and noninstitutionalized  schools were included in the study”  “Based on the previous literature and using the  sample size formula, a total of 350 school children” |
| **Q5** | Indicate if evaluators of subjective components of study were masked to other aspects of the status of the participants. | 1= evaluator masked  2= not masked  **3= unclear /not mentioned** | **Comment**: The caries assessor can be blinded about the multimedia habits and vice versa. But no mentioning of this was reported. |
| **Q6** | Describe any assessments undertaken for quality assurance purposes (e.g., test/retest of primary outcome measurements) | **1= exposure & outcome tools validated/examiner-kappa-score reported)**  2= not done  3= unclear /partially done | **“**Inter-examiner reliability was evaluated with Kappa statistic which was found to be 0.7.” |
| **Q7** | Explain any patient exclusions from analysis | 1=mentioned clearly  **2=not mentioned**  3=unclear information  4=NA | “Multimedia habits for all institutionalized school children were similar and hence the association  with dental caries was not possible.”  **Comment:** One whole outcome (multimedia habits) of institutionalized group was not reported. If association would be meaningless, that does not mean not to mention the results without further statistical analysis. |
| **Q8** | Describe how confounding was assessed and/or controlled. | 1=mentioned (design/analysis)  **2=not done**  3=unclear /not mentioned |  |
| **Q9** | If applicable, explain how missing data were handled in the analysis | 1= clearly mentioned  **2= no information**  3= unclear / insufficient info | **Comment**: no missing data; So, low risk of bias |
| **Q10** | Summarize patient response rates and completeness of data collection | 1=mentioned & above 80%  **2=not mentioned**  3=unclear information | **Comment:** The sample size was fulfilled so low risk. |
| **Q11** | Clarify what follow-up, if any, was expected and the percentage of patients for which incomplete data or follow-up was obtained | 1= clearly mentioned  2= not mentioned  **3= unclear / not applicable** | NA |

1. **(Meshki et al., 2022)**

| **No.** | **Description of question** | **Criteria** | **Comment from the record (verbatim)** |
| --- | --- | --- | --- |
| **Q1** | Define the source of information  (Survey, record review) | **1= from survey**  2= not mentioned  3= records/ unclear info | “The present cross-sectional study evaluated children from orphanages belonging to ….” |
| **Q2** | List inclusion and exclusion criteria for exposed and unexposed subjects (cases and controls) or refer to previous publications | **1= clearly mentioned**  2= no information  3= unclear / insufficient info | “The exclusion criteria were ……….. ” |
| **Q3** | Indicate time period used for identifying subjects | **1= time period given**  2= no info given | “The study was con­ducted from July 2020 to January 2021.” |
| **Q4** | Indicate whether or not subjects were consecutive if not population based. | **1= representative**  2= not representative (convenience/ not randomly selected)  3= no clear info | “A simple randomization method and a table of random num­bers were used. Every school child was assigned a number. If the selected number was even, the school child was allocated to the study group, and if it was odd, the school child was excluded from the study.” |
| **Q5** | Indicate if evaluators of subjective components of study were masked to other aspects of the status of the participants. | 1= evaluator masked  **2= not masked**  3= unclear /not mentioned | **Comment:** Masking is not applicable  So **low risk of bias** |
| **Q6** | Describe any assessments undertaken for quality assurance purposes (e.g., test/retest of primary outcome measurements) | **1= exposure & outcome tools validated/examiner-kappa-score reported)**  2= not done  3= unclear /partially done | **“**The reliability of the examiner was eval­uated by test re-test with the re-examination of 11% of the sam­ples.” |
| **Q7** | Explain any patient exclusions from analysis | 1=mentioned clearly  2=not mentioned  3=unclear information  **4=NA** | **Comment:** No patients excluded; So, low risk of bias |
| **Q8** | Describe how confounding was assessed and/or controlled. | **1=mentioned (design/analysis)**  2=not done  3=unclear /not mentioned | **Comment:** Subgroup analysis of results regarding the gender. |
| **Q9** | If applicable, explain how missing data were handled in the analysis | 1= clearly mentioned  **2= no information**  3= unclear / insufficient info | **Comment**: no missing data; So, low risk of bias |
| **Q10** | Summarize patient response rates and completeness of data collection | 1=mentioned & above 80%  **2=not mentioned**  3=unclear information | **Comment:** The sample size was fulfilled so low risk. |
| **Q11** | Clarify what follow-up, if any, was expected and the percentage of patients for which incomplete data or follow-up was obtained | 1= clearly mentioned  2= not mentioned  **3= unclear / not applicable** | NA |

1. **(Mohan et al., 2014)**

| **No.** | **Description of question** | **Criteria** | **Comment from the record (verbatim)** |
| --- | --- | --- | --- |
| **Q1** | Define the source of information  (Survey, record review) | **1= from survey**  2= not mentioned  3= records/ unclear info | “All intraoral examinations were done using ….” |
| **Q2** | List inclusion and exclusion criteria for exposed and unexposed subjects (cases and controls) or refer to previous publications | **1= clearly mentioned**  2= no information  3= unclear / insufficient info | “Inclusion criteria were ……….. ” |
| **Q3** | Indicate time period used for identifying subjects | 1= time period given  **2= no info given** |  |
| **Q4** | Indicate whether or not subjects were consecutive if not population based. | **1= representative**  2= not representative (convenience/ not randomly selected)  3= no clear info | “ Three orphanages were selected.”  **Comment:** random selection of the orphanages, but sample size calculation was not performed. |
| **Q5** | Indicate if evaluators of subjective components of study were masked to other aspects of the status of the participants. | 1= evaluator masked  **2= not masked**  3= unclear /not mentioned | **Comment:** Masking is not applicable  So **low risk of bias** |
| **Q6** | Describe any assessments undertaken for quality assurance purposes (e.g., test/retest of primary outcome measurements) | **1= exposure & outcome tools validated/examiner-kappa-score reported)**  2= not done  3= unclear /partially done | “Clinical diagnostic criteria proposed by World Health Organization [3], Dentition Status and Treatment needs index [4] and Community Periodontal index for treatment needs [5]” |
| **Q7** | Explain any patient exclusions from analysis | 1=mentioned clearly  2=not mentioned  3=unclear information  **4=NA** | **Comment:** No patients excluded; So, low risk of bias |
| **Q8** | Describe how confounding was assessed and/or controlled. | **1=mentioned (design/analysis)**  2=not done  3=unclear /not mentioned | **Comment:** Subgroup analysis of results regarding the gender. |
| **Q9** | If applicable, explain how missing data were handled in the analysis | 1= clearly mentioned  **2= no information**  3= unclear / insufficient info | **Comment**: no missing data; So, low risk of bias |
| **Q10** | Summarize patient response rates and completeness of data collection | 1=mentioned & above 80%  **2=not mentioned**  3=unclear information | **Comment**: As there is no sample size calculation and no specification of the capacity of the orphanages, the response rate can not be concluded. |
| **Q11** | Clarify what follow-up, if any, was expected and the percentage of patients for which incomplete data or follow-up was obtained | 1= clearly mentioned  2= not mentioned  **3= unclear / not applicable** | NA |

1. **(Pavithran et al., 2009)**

| **No.** | **Description of question** | **Criteria** | **Comment from the record (verbatim)** |
| --- | --- | --- | --- |
| **Q1** | Define the source of information  (Survey, record review) | **1= from survey**  2= not mentioned  3= records/ unclear info | “On the day of interview and examination, ….” |
| **Q2** | List inclusion and exclusion criteria for exposed and unexposed subjects (cases and controls) or refer to previous publications | **1= clearly mentioned**  2= no information  3= unclear / insufficient info | “Based on the selection criteria” |
| **Q3** | Indicate time period used for identifying subjects | **1= time period given**  2= no info given | “Data were collected over a period of 5 months, from March 2014 to July 2014” |
| **Q4** | Indicate whether or not subjects were consecutive if not population based. | **1= representative**  2= not representative (convenience/ not randomly selected)  3= no clear info | “Previous studies[14],[15] were considered for calculating the sample size”  “As a sample size of 210 orphans was to be included in the study, 40 orphanages were selected through simple random sampling technique (lottery method)” |
| **Q5** | Indicate if evaluators of subjective components of study were masked to other aspects of the status of the participants. | 1= evaluator masked  **2= not masked**  3= unclear /not mentioned | **Comment:** Masking is not applicable  So **low risk of bias** |
| **Q6** | Describe any assessments undertaken for quality assurance purposes (e.g., test/retest of primary outcome measurements) | **1= exposure & outcome tools validated/examiner-kappa-score reported)**  2= not done  3= unclear /partially done | “One examiner (VKP) was trained and calibrated by clinically experienced faculty of the department. The examinerpracticed and repeated the recordings on 20 children; intraexaminer reliability was found to be *k* = 0.85”. |
| **Q7** | Explain any patient exclusions from analysis | 1=mentioned clearly  2=not mentioned  3=unclear information  **4=NA** | **Comment:** No patients excluded; So, low risk of bias |
| **Q8** | Describe how confounding was assessed and/or controlled. | 1=mentioned (design/analysis)  **2=not done**  3=unclear /not mentioned |  |
| **Q9** | If applicable, explain how missing data were handled in the analysis | 1= clearly mentioned  **2= no information**  3= unclear / insufficient info | **Comment**: no missing data; So, low risk of bias |
| **Q10** | Summarize patient response rates and completeness of data collection | 1=mentioned & above 80%  **2=not mentioned**  3=unclear information | **Comment:** The sample size was fulfilled so low risk. |
| **Q11** | Clarify what follow-up, if any, was expected and the percentage of patients for which incomplete data or follow-up was obtained | 1= clearly mentioned  2= not mentioned  **3= unclear / not applicable** | NA |

1. **(Rimaviciute et al., 2019)**

| **No.** | **Description of question** | **Criteria** | **Comment from the record (verbatim)** |
| --- | --- | --- | --- |
| **Q1** | Define the source of information  (Survey, record review) | **1= from survey**  2= not mentioned  3= records/ unclear info | “Clinical examination of the oral cavity was  performed in….” |
| **Q2** | List inclusion and exclusion criteria for exposed and unexposed subjects (cases and controls) or refer to previous publications | **1= clearly mentioned**  2= no information  3= unclear / insufficient info | “Only  healthy 12-year and 15-year-old adolescents without any mental and physical disabilities were included into the study” |
| **Q3** | Indicate time period used for identifying subjects | **1= time period given**  2= no info given | “…between February and September of 2019” |
| **Q4** | Indicate whether or not subjects were consecutive if not population based. | **1= representative**  2= not representative (convenience/ not randomly selected)  3= no clear info | “All adolescents residing  at care homes at that time were examined.  Control group was selected at random from  schools of the same regions. One school from every  region (3 in total) was selected by computer and all 12 and 15 year-old adolescents were examined who were present at school at that time.”  **Comment:** No sample size calculation. |
| **Q5** | Indicate if evaluators of subjective components of study were masked to other aspects of the status of the participants. | 1= evaluator masked  **2= not masked**  3= unclear /not mentioned | **Comment:** Masking is not applicable  So **low risk of bias** |
| **Q6** | Describe any assessments undertaken for quality assurance purposes (e.g., test/retest of primary outcome measurements) | **1= exposure & outcome tools validated/examiner-kappa-score reported)**  2= not done  3= unclear /partially done | “The diagnostic threshold was according the WHO.  Dental plaque was evaluated by the Plaque Index (Silness and Loe, 1964).”  “An oral examination was performed by the same investigator.” |
| **Q7** | Explain any patient exclusions from analysis | **1=mentioned clearly**  2=not mentioned  3=unclear information  4=NA | “The study excluded subjects with  values of all variables greater than Q3+  3×IQR. The data of these subjects were  excluded and not used in statistical analysis  (one subject of the study and four subjects  of the control groups).” |
| **Q8** | Describe how confounding was assessed and/or controlled. | **1=mentioned (design/analysis)**  2=not done  3=unclear /not mentioned | **Comment:** Subgroup analysis of results regarding the age and gender. |
| **Q9** | If applicable, explain how missing data were handled in the analysis | 1= clearly mentioned  **2= no information**  3= unclear / insufficient info | **Comment**: no missing data; So, low risk of bias |
| **Q10** | Summarize patient response rates and completeness of data collection | 1=mentioned & above 80%  **2=not mentioned**  3=unclear information | **Comment**: As there is no sample size calculation and no specification of the capacity of the orphanages, the response rate can not be concluded. |
| **Q11** | Clarify what follow-up, if any, was expected and the percentage of patients for which incomplete data or follow-up was obtained | 1= clearly mentioned  2= not mentioned  **3= unclear / not applicable** | NA |

1. **(Shah et al., 2016)**

AHRQ tool **for cross-sectional (yes/No/ Unclear)**

| **No.** | **Description of question** | **Criteria** | **Comment from the record (verbatim)** |
| --- | --- | --- | --- |
| **Q1** | Define the source of information  (Survey, record review) | **1= from survey**  2= not mentioned  3= records/ unclear info | “examination of subjects was conducted at respective orphanages…” |
| **Q2** | List inclusion and exclusion criteria for exposed and unexposed subjects (cases and controls) or refer to previous publications | **1= clearly mentioned**  2= no information  3= unclear / insufficient info | ” The inclusion criteria were that…..” |
| **Q3** | Indicate time period used for identifying subjects | **1= time period given**  2= no info given | “over a period of  3 months from August 2012 to October 2012.” |
| **Q4** | Indicate whether or not subjects were consecutive if not population based. | **1= representative**  2= not representative (convenience/ not randomly selected)  3= no clear info | “The total population of the orphanages that gave positive Consent…..” |
| **Q5** | Indicate if evaluators of subjective components of study were masked to other aspects of the status of the participants. | 1= evaluator masked  **2= not masked**  3= unclear /not mentioned | **Comment:** Masking is not applicable  So **low risk of bias** |
| **Q6** | Describe any assessments undertaken for quality assurance purposes (e.g., test/retest of primary outcome measurements) | **1= exposure & outcome tools validated/examiner-kappa-score reported)**  2= not done  3= unclear /partially done | ” in order to limit the intraexaminer  variability. The agreement for all the assessments was in  the range of 85 to 95%.” |
| **Q7** | Explain any patient exclusions from analysis | **1=mentioned clearly**  2=not mentioned  3=unclear information  4=NA | “There were only a small number of children below 6 years of age who satisfied the inclusion criteria and less than 25 children above 13 years of age residing in these orphanages. Most of these were not present at the time of examination.”  **Comment:** No patients were excluded |
| **Q8** | Describe how confounding was assessed and/or controlled. | **1=mentioned (design/analysis)**  2=not done  3=unclear /not mentioned | **Comment:** testing of all variables in relation to caries, age and gender. |
| **Q9** | If applicable, explain how missing data were handled in the analysis | 1= clearly mentioned  **2= no information**  3= unclear / insufficient info | **Comment**: no missing data; So, low risk of bias |
| **Q10** | Summarize patient response rates and completeness of data collection | 1=mentioned & above 80%  **2=not mentioned**  3=unclear information | **Comment**: As there is no sample size calculation and no specification of the capacity of the orphanages, the response rate can not be concluded. |
| **Q11** | Clarify what follow-up, if any, was expected and the percentage of patients for which incomplete data or follow-up was obtained | 1= clearly mentioned  2= not mentioned  **3= unclear / not applicable** | Not applicable |

1. **(Shanthi et al., 2017)**

AHRQ tool **for cross-sectional (yes/No/ Unclear)**

| **No.** | **Description of question** | **Criteria** | **Comment from the record (verbatim)** |
| --- | --- | --- | --- |
| **Q1** | Define the source of information  (Survey, record review) | **1= from survey**  2= not mentioned  3= records/ unclear info | “prior to examination from orphanage  officials. A self-administered structured questionnaire…” |
| **Q2** | List inclusion and exclusion criteria for exposed and unexposed subjects (cases and controls) or refer to previous publications | **1= clearly mentioned**  2= no information  3= unclear / insufficient info |  |
| **Q3** | Indicate time period used for identifying subjects | 1= time period given  **2= no info given** |  |
| **Q4** | Indicate whether or not subjects were consecutive if not population based. | 1= representative  **2= not representative (convenience/ not randomly selected)**  3= no clear info | **Comment:** Available participants were selected randomly. (but no sample size calculation |
| **Q5** | Indicate if evaluators of subjective components of study were masked to other aspects of the status of the participants. | 1= evaluator masked  **2= not masked**  3= unclear /not mentioned | **Comment:** Masking is not applicable  So **low risk of bias** |
| **Q6** | Describe any assessments undertaken for quality assurance purposes (e.g., test/retest of primary outcome measurements) | **1= exposure & outcome tools validated/examiner-kappa-score reported)**  2= not done  3= unclear /partially done | “The WHO Oral Health Assessment form11 was used to record primary and permanent dentition status and treatment needs of the study population” |
| **Q7** | Explain any patient exclusions from analysis | 1=mentioned clearly  2=not mentioned  3=unclear information  **4=NA** | **Comment:** No patients excluded; So, low risk of bias |
| **Q8** | Describe how confounding was assessed and/or controlled. | 1=mentioned (design/analysis)  **2=not done**  3=unclear /not mentioned |  |
| **Q9** | If applicable, explain how missing data were handled in the analysis | 1= clearly mentioned  **2= no information**  3= unclear / insufficient info | **Comment**: no missing data; So, low risk of bias |
| **Q10** | Summarize patient response rates and completeness of data collection | 1=mentioned & above 80%  **2=not mentioned**  3=unclear information | **Comment**: As there is no sample size calculation and no specification of the capacity of the orphanages, the response rate can not be concluded. |
| **Q11** | Clarify what follow-up, if any, was expected and the percentage of patients for which incomplete data or follow-up was obtained | 1= clearly mentioned  2= not mentioned  **3= unclear / not applicable** | Not applicable |

1. **(Shuangjiao et al., 2014)**

AHRQ tool **for cross-sectional (yes/No/ Unclear)**

| **No.** | **Description of question** | **Criteria** | **Comment from the record (verbatim)** |
| --- | --- | --- | --- |
| **Q1** | Define the source of information  (Survey, record review) | **1= from survey**  2= not mentioned  3= records/ unclear info | “ 2 dental professionals participated in the examination, and 2 nursing staff assisted in filling out the form …” |
| **Q2** | List inclusion and exclusion criteria for exposed and unexposed subjects (cases and controls) or refer to previous publications | **1= clearly mentioned**  2= no information  3= unclear / insufficient info | ” Exclusion criteria：  1) Children with severe disabilities  2) Children with systemic diseases  3) The age of children is between 0~3 years old …..” |
| **Q3** | Indicate time period used for identifying subjects | 1= time period given  **2= no info given** |  |
| **Q4** | Indicate whether or not subjects were consecutive if not population based. | **1= representative**  2= not representative (convenience/ not randomly selected)  3= no clear info | “from a children's welfare home in Jiangbei District, Chongqing (the welfare home is the largest child welfare institution in Chongqing City, and the orphans come from multiple districts and counties in Chongqing).…..” |
| **Q5** | Indicate if evaluators of subjective components of study were masked to other aspects of the status of the participants. | 1= evaluator masked  **2= not masked**  3= unclear /not mentioned | **Comment:** Masking is not applicable  So **low risk of bias** |
| **Q6** | Describe any assessments undertaken for quality assurance purposes (e.g., test/retest of primary outcome measurements) | **1= exposure & outcome tools validated/examiner-kappa-score reported)**  2= not done  3= unclear /partially done | ” The Kappa value of each inspector was above 0.8. ” |
| **Q7** | Explain any patient exclusions from analysis | **1=mentioned clearly**  2=not mentioned  3=unclear information  4=NA | **Comment**: No patients excluded; So, low risk of bias. |
| **Q8** | Describe how confounding was assessed and/or controlled. | **1=mentioned (design/analysis)**  2=not done  3=unclear /not mentioned | **Comment:** Subgroup analysis of results regarding the age and gender. |
| **Q9** | If applicable, explain how missing data were handled in the analysis | **1= clearly mentioned**  2= no information  3= unclear / insufficient info | **Comment**: no missing data; So, low risk of bias. |
| **Q10** | Summarize patient response rates and completeness of data collection | **1=mentioned & above 80%**  2=not mentioned  3=unclear information | **Comment**: Although there is no sample size calculation, the capacity of the orphanage was specified (503). All eligible children were examined (317 out of 503) |
| **Q11** | Clarify what follow-up, if any, was expected and the percentage of patients for which incomplete data or follow-up was obtained | 1= clearly mentioned  2= not mentioned  **3= unclear / not applicable** | Not applicable |

1. **(Suresan et al., 2021)**

AHRQ tool **for cross-sectional (yes/No/ Unclear)**

| **No.** | **Description of question** | **Criteria** | **Comment from the record (verbatim)** |
| --- | --- | --- | --- |
| **Q1** | Define the source of information  (Survey, record review) | **1= from survey**  2= not mentioned  3= records/ unclear info |  |
| **Q2** | List inclusion and exclusion criteria for exposed and unexposed subjects (cases and controls) or refer to previous publications | **1= clearly mentioned**  2= no information  3= unclear / insufficient info | “Orphans who refused to participate and those with …. were excluded from the….” |
| **Q3** | Indicate time period used for identifying subjects | **1= time period given**  2= no info given | “from March  2019 to September 2019” |
| **Q4** | Indicate whether or not subjects were consecutive if not population based. | **1= representative**  2= not representative (convenience/ not randomly selected)  3= no clear info | “A universal  sampling technique”  **Comment:** but no sample size calculation. |
| **Q5** | Indicate if evaluators of subjective components of study were masked to other aspects of the status of the participants. | 1= evaluator masked  **2= not masked**  3= unclear /not mentioned | **Comment:** Masking is not applicable  So **low risk of bias** |
| **Q6** | Describe any assessments undertaken for quality assurance purposes (e.g., test/retest of primary outcome measurements) | **1= exposure & outcome tools validated/examiner-kappa-score reported)**  2= not done  3= unclear /partially done | “The intra-examiner reliability kappa value was 0.89 indicating high reliability” |
| **Q7** | Explain any patient exclusions from analysis | 1=mentioned clearly  2=not mentioned  3=unclear information  **4=NA** | **Comment:** No patients excluded; So, low risk of bias |
| **Q8** | Describe how confounding was assessed and/or controlled. | **1=mentioned (design/analysis)**  2=not done  3=unclear /not mentioned | **Comment:** Subgroup analysis of results regarding the age and gender. |
| **Q9** | If applicable, explain how missing data were handled in the analysis | 1= clearly mentioned  **2= no information**  3= unclear / insufficient info | **Comment**: no missing data; So, low risk of bias |
| **Q10** | Summarize patient response rates and completeness of data collection | **1=mentioned & above 80%**  2=not mentioned  3=unclear information | A coverage rate of 94.5% was recorded for this study. |
| **Q11** | Clarify what follow-up, if any, was expected and the percentage of patients for which incomplete data or follow-up was obtained | 1= clearly mentioned  2= not mentioned  **3= unclear / not applicable** | Not applicable |

1. **(THETAKALA et al., 2017)**

| **No.** | **Description of question** | **Criteria** | **Comment from the record (verbatim)** |
| --- | --- | --- | --- |
| **Q1** | Define the source of information  (Survey, record review) | **1= from survey**  2= not mentioned  3= records/ unclear info | “…… on children from eight orphanages and seven government schools” |
| **Q2** | List inclusion and exclusion criteria for exposed and unexposed subjects (cases and controls) or refer to previous publications | **1= clearly mentioned**  2= no information  3= unclear / insufficient info | “Children with any systemic diseases and mental disability were excluded” |
| **Q3** | Indicate time period used for identifying subjects | **1= time period given**  2= no info given | ““was undertaken over a period of six months from March to August 2015” |
| **Q4** | Indicate whether or not subjects were consecutive if not population based. | **1= representative**  2= not representative (convenience/ not randomly selected)  3= no clear info | “14 registered orphanages, out of which, two were for physically challenged children (one for blind and another for deaf and dumb children). Among the remaining, four orphanages declined to grant permission for study due to various reasons such as having their own dentist, lack of interest and financial aspects. “Finally, eight orphanages consisting of 484 inmates ”  orphanages, all the available children residing in these orphanages were considered.”  **Comment:** and sample size calculation was performed and reported in details. |
| **Q5** | Indicate if evaluators of subjective components of study were masked to other aspects of the status of the participants. | 1= evaluator masked  **2= not masked**  3= unclear /not mentioned | **Comment:** Masking is not applicable  So **low risk of bias** |
| **Q6** | Describe any assessments undertaken for quality assurance purposes (e.g., test/retest of primary outcome measurements) | **1= exposure & outcome tools validated/examiner-kappa-score reported)**  2= not done  3= unclear /partially done | “Training and calibration of the investigator” “The intra-examiner reliability for dentition status (untreated carious lesions) was assessed. The kappa coefficient value for intra-examiner reliability was found to be 0.94.” |
| **Q7** | Explain any patient exclusions from analysis | 1=mentioned clearly  2=not mentioned  3=unclear information  **4=NA** | **Comment:** No patients excluded; So, low risk of bias |
| **Q8** | Describe how confounding was assessed and/or controlled. | **1=mentioned (design/analysis)**  2=not done  3=unclear /not mentioned | **Comment:** Subgroup analysis of results regarding the gender. |
| **Q9** | If applicable, explain how missing data were handled in the analysis | 1= clearly mentioned  **2= no information**  3= unclear / insufficient info | **Comment**: no missing data; So, low risk of bias |
| **Q10** | Summarize patient response rates and completeness of data collection | 1=mentioned & above 80%  **2=not mentioned**  3=unclear information | **Comment:** The sample size was fulfilled so low risk. |
| **Q11** | Clarify what follow-up, if any, was expected and the percentage of patients for which incomplete data or follow-up was obtained | 1= clearly mentioned  2= not mentioned  **3= unclear / not applicable** | Not applicable |

1. **(Xu et al., 2021)**

| **No.** | **Description of question** | **Criteria** | **Comment from the record (verbatim)** |
| --- | --- | --- | --- |
| **Q1** | Define the source of information  (Survey, record review) | **1= from survey**  2= not mentioned  3= records/ unclear info | “Visual and exploratory examinations were performed” |
| **Q2** | List inclusion and exclusion criteria for exposed and unexposed subjects (cases and controls) or refer to previous publications | **1= clearly mentioned**  2= no information  3= unclear / insufficient info | “The inclusion criteria for……” |
| **Q3** | Indicate time period used for identifying subjects | **1= time period given**  2= no info given | “From September  15, 2020 to December 15, 2020” |
| **Q4** | Indicate whether or not subjects were consecutive if not population based. | **1= representative**  2= not representative (convenience/ not randomly selected)  3= no clear info | “We selected schools and orphanages in Fuyang City by lottery, and all children from the selected institutions were regarded as a whole. After all the children were numbered, random numbers were generated by a random number generators, and then the children corresponding to the random numbers were studied. The sample size calculation formula:” |
| **Q5** | Indicate if evaluators of subjective components of study were masked to other aspects of the status of the participants. | 1= evaluator masked  **2= not masked**  3= unclear /not mentioned | **Comment:** Masking is not applicable  So **low risk of bias** |
| **Q6** | Describe any assessments undertaken for quality assurance purposes (e.g., test/retest of primary outcome measurements) | **1= exposure & outcome tools validated/examiner-kappa-score reported)**  2= not done  3= unclear /partially done | “The standard consistency test results before the survey were reliable (Kappa value > 0.8).” |
| **Q7** | Explain any patient exclusions from analysis | 1=mentioned clearly  2=not mentioned  3=unclear information  **4=NA** | **Comment:** No patients excluded; So, low risk of bias |
| **Q8** | Describe how confounding was assessed and/or controlled. | **1=mentioned (design/analysis)**  2=not done  3=unclear /not mentioned | **Comment:** Subgroup analysis of results regarding the age and gender. |
| **Q9** | If applicable, explain how missing data were handled in the analysis | 1= clearly mentioned  **2= no information**  3= unclear / insufficient info | **Comment**: no missing data; So, low risk of bias |
| **Q10** | Summarize patient response rates and completeness of data collection | 1=mentioned & above 80%  **2=not mentioned**  3=unclear information | **Comment:** The sample size was fulfilled so low risk. |
| **Q11** | Clarify what follow-up, if any, was expected and the percentage of patients for which incomplete data or follow-up was obtained | 1= clearly mentioned  2= not mentioned  **3= unclear / not applicable** | Not applicable |

**References:**

Abedassar, S., Malek-mohammadi, T., Dehesh, T., & Dahesh, S. (2022). Oral health status and oral hygiene behaviour of orphan children: A survey in support centers in Kerman City, Iran, in 2019. *J Oral Health Oral Epidemiol 2022;*, *11*(1), 32–39. https://doi.org/10.22122/johoe.v11i1.1221

Agarwalla, S., Chandra, B., Santra, A., & Kundu, G. K. (2022). Impact of Intelligence Quotient (IQ) on Dental Caries amongst Socially Handicapped Orphan Children and Children Living with Their Parents. *International Journal of Clinical Pediatric Dentistry*, *15*(2), 0–3.

Al‑maweri, S. A., Al‑soneidar, walid A., & Halboub, Es. S. (2014). Oral lesions and dental status among institutionalized orphans in Yemen : A matched case‑control study. *Contemporary Clinical Dentistry*, *5*(1), 81–84. https://doi.org/10.4103/0976-237X.128673

Babu, K. L. G., & Kavyashree, G. H. (2021). Evaluation of Oral Health ‑ Related Quality of Life among Institutionalized Orphan Children. *Journal of Forensic Scince and Medicine*, *7*, 117–122. https://doi.org/10.4103/jfsm.jfsm

CHANDRAN, T. (2017). *ASSOCIATION OF SELF CONCEPT AND ORAL HEALTH STATUS AMONG 12 – 17 YEAR OLDS RESIDING IN ORPHANAGES IN BENGALURU*.

Chandran, T., Ravindranath, N. S., Raju, R., Samuel, S., James, J. M., & Mistry, F. (2021). Association of Self-Concept with Oral Health Status among Children Residing in Orphanages in Bengaluru. *Journal of Evolution of Medical and Dental Sciences*, *10*(33), 2795–2798. https://doi.org/10.14260/jemds/2021/570

Gaytry, S. S. (2018). *Comparison of caries experience with the salivary levels of streprococcus mutans and Lactobacilli in 8-14 years old children between institutionalized (orphanage) children and school going children in Namakkal district, Tamilnadu*.

Kavayashree, G., & Babu, K. L. G. (2019). Assessment of Oral Health Status of Children Living in Orphanages of Hassan City , India. *Journal of Indian Association of Public Health Dentostry*, *17*, 201–205. https://doi.org/10.4103/jiaphd.jiaphd

Khattab, N. M. A., & Abd-ElSabour, M. A. A. (2023). Assessment of dental caries among a group of institutionalized orphan children compared to parented school children : case–control study. *BMC Oral Health*, *23*(202). https://doi.org/10.1186/s12903-023-02915-1

Khedekar, M., Suresh, K. V., Parkar, M. I., Malik, N., Patil, S., Taur, S., & Pradhan, D. (2015). Implementation of oral health education to orphan children. *Journal of the College of Physicians and Surgeons Pakistan*, *25*(12), 856–859. https://doi.org/12.2015/JCPSP.856859

Kong, L., Qin, D., & Wang, J. (2017). Oral Health Status in Chongqing Orphans at 3-5 and 12-15 Years Old: A Sampling Survey. *EC Dental Science*, *10*(1), 1–8. https://cran.r-project.org/package=sampling

Marasouli, P., Khoshrou, M. M., Hoseinzadeh, M., & Niknejad, E. (2016). [SURVEY OF DMFT AND DMFT INDICES IN URMIA ORPHANAGES IN 6-18 YEAR-OLD INDIVIDUALS IN 2014]. *The Journal of Urmia University of Medical Sciences*, *27*(8), 729–734.

Mehta, V., Shetiya, S. H., Kakodkar, P., Rajpurohit, L., & Kumbhalwar, A. (2020). Association between Dental Caries and Multimedia Habits amongst Institutionalized and Non-Institutionalized Children. *European Journal of Molecular and Clinical Medicine*, *7*(11), 7277–7283.

Meshki, R., Basir, L., Motaghi, S., & Kazempour, M. (2022). Oral health status among orphan and non-orphan children in Mashhad : a case-control study. *Journal of Medicine and Life*, *15*(9), 1198–1201. https://doi.org/10.25122/jml-2021-0127

Mohan, A., Misra, N., Umapathy, D., Kumar, S., Srivastav, D., & Mohan, U. (2014). Oral and dental health status in orphan children of Lucknow. *Indian Journal of Community Health*, *26*(2), 170–173.

Pavithran, V., Murali, R., Krishna, M., Shamala, A., Yalamalli, M., Kumar, A. V, & Raina, R. (2009). Impact of oral diseases on daily activities among 12- to 15-year-old institutionalized orphan and non-orphan children in Bengaluru city: A cross-sectional analytical study. *Indian Journal of Dental Research : Official Publication of Indian Society for Dental Research*, *31*(2), 396–402.

Rimaviciute, A., Domeikaite, M., Mazecaite-Vaitilaviciene, L., Bendinskaite, R., & Puriene, A. (2019). Oral health of 12 and 15 year-old adolescents living in the social care homes in south of Lithuania. *Stomatologija*, *21*(4), 119–124.

Shah, A. F., Tangade, P., Ravishankar, T., Tirth, A., Pal, S., & Batra, M. (2016). Dental Caries Status of Institutionalized Orphan Children from Jammu and Kashmir , India. *Int j Clin Pediatr Denr*, *9*(4), 364–371.

Shanthi, M., Goud, E. S. S. V. S. S., Kumar, G. P., Rajguru, J. P., Ratnasothy, S., & Ealla, K. K. R. K. (2017). Risk factors and treatment needs among orphan school children. *Journal of Contemporary Dental Practice*, *18*(10), 893–898. https://doi.org/10.5005/jp-journals-10024-2145

Shuangjiao, G., Juhong, L., Weiwen, Q., Yuhong, W., Xuehua, Z., Yuzhi, W., Menghong, W., Weiwen, Q., Yuhong, W., Xuehua, Z., & Yuzhi, W. (2014). [Oral health status of 4-17-year-old orphan children and adolescents of Chongqing] Chóngqìng shì 4~17 suì gū’ér értóng qīngshàonián kǒuqiāng jiànkāng zhuàngkuàng diàochá. *West China Journal of Stomatology*, *32*(4), 378–381. https://doi.org/10.7518/hxkq.2014.04.014

Suresan, V., Jha, K., Diptajit, D., Sourav, S., Jalaluddin, M., & Fatima, A. (2021). Dental caries experience and oral hygiene status among institutionalized orphans of bhubaneswar city, odisha: A comprehensive dental healthcare program outcome. *World Journal of Dentistry*, *12*(2), 131–137. https://doi.org/10.5005/jp-journals-10015-1810

THETAKALA, R. K., SUNITHA, S., CHANDRASHEKAR, B., SHARMA, Pri., KRUPA, N., & SRILATHA, Y. (2017). Periodontal and Dentition Status among Orphans and Children with Parents in Mysore City , India : A Comparative Study. *Journal of Clinical and Diagnostic Research*, *11*(4), ZC115–ZC118. https://doi.org/10.7860/JCDR/2017/25655.9748

Xu, J., Zhu, Y., Wang, C., & Wang, D. (2021). The Dental Health of Orphan and Non-orphan Children in Fuyang City , China. *Oral Health & Preventive Dentistry*, *19*, 523–527. https://doi.org/10.3290/j.ohpd.b2182947

Zeng, X., Zhang, Y., Kwong, J. S. W., Zhang, C., Li, S., & Sun, F. (2015). The methodological quality assessment tools for preclinical and clinical studies , systematic review and meta-analysis , and clinical practice guideline : a systematic review. *Journal of Evidence-Based Medicine*, *8*, 2–10. https://doi.org/10.1111/jebm.12141
